# Baicalin nanodelivery system based on functionalized metal-organic framework for targeted therapy of osteoarthritis by modulating macrophage polarization

**DOI:** 10.1186/s12951-024-02494-5

**Published:** 2024-05-09

**Authors:** Lanli Huang, Yi Yao, Zhuren Ruan, Shengqing Zhang, Xianjing Feng, Chun Lu, Jinmin Zhao, Feiying Yin, Cunwei Cao, Li Zheng

**Affiliations:** 1 Guangxi Engineering Center in Biomedical Material for Tissue and Organ Regeneration, Collaborative Innovation Centre of Regenerative Medicine and Medical BioResource Development and Application Co-constructed By the Province and Ministry, Guangxi Key Laboratory of Regenerative Medicine, The First Affiliated Hospital of Guangxi Medical University, Nanning, 530021 China; 2 Life Sciences Institute, Guangxi Medical University, Nanning, 530021 China; 3 Department of Dermatology and Venereology, The First Affiliated Hospital of Guangxi Medical University, Nanning, Guangxi China; 4 Pharmaceutical College, Guangxi Medical University, Nanning, 530021 China; 5 School of Materials and Environment, Guangxi Minzu University, Nanning, 53000 China

**Keywords:** M1-macrophage-targeting, Nano-delivery system, Controlled release, Baicalin, High drug loading, Osteoarthritis

## Abstract

Intra-articular drugs used to treat osteoarthritis (OA) often suffer from poor pharmacokinetics and stability. Nano-platforms as drug delivery systems for drug delivery are promising for OA therapy. In this study, we reported an M1 macrophage-targeted delivery system Bai@FA-UIO-66-NH_2_ based on folic acid (FA) -modified metal-organic framework (MOF) loaded with baicalin (Bai) as antioxidant agent for OA therapy. With outstanding biocompatibility and high drug loading efficiency, Bai@FA-UIO-66-NH_2_ could be specifically uptaken by LPS-induced macrophages to serve as a potent ROS scavenger, gradually releasing Bai at the subcellular level to reduce ROS production, modulate macrophage polarization to M2, leading to alleviation of synovial inflammation in OA joints. The synergistic effect of Bai@FA-UIO-66-NH_2_ on macrophage polarization and ROS scavenging significantly improved the therapeutic efficacy of OA, which may provide a new insight into the design of OA precision therapy.

**Supplementary Information:**

The online version contains supplementary material available at 10.1186/s12951-024-02494-5.

## Introduction

Osteoarthritis (OA), characterized by high morbidity and disability, is a common chronic joint disease that endangers public health worldwide [1, 2]. The pathogenesis of OA is extremely complex, and there is currently no effective treatment strategy. Accumulative evidence [3–5] has shown that synovial inflammation is a major contributor to OA. Macrophages of the synovial membrane secrete pro-inflammatory factors, accelerating cartilage destruction [5–9]. Furthermore, the proportion of M1/M2 macrophages was unbalanced in OA patients’ synovial membranes and peripheral blood [10], contributing to the severity of OA. Zhang Yanhai et al. [11] confirmed that the inability of macrophages to transition from M1 to M2 is another major factor accelerating OA inflammation. In addition, excessive subcellular reactive oxygen species (ROS) produced in the joint cavity induced the polarization of macrophage M1, which mediated the production and release of pro-inflammatory cytokines in peripheral tissue, thus aggravating the inflammatory response [12–14]. Therefore, regulating ROS levels at the cellular level and further macrophage repolarization (M1-M2) are potential targets for OA therapy.

Long-term use of OA clinical therapeutic drugs led to adverse effects such as host rejection. Traditional Chinese medicine has immunomodulatory, antioxidant and cell-protective properties with fewer side effects. Baicalin (Bai), derived from cultivated Scutellaria baicalensis, is a traditional Chinese medicine flavonoid with excellent anti-inflammatory and antioxidant properties [15, 16] and has been widely used for anti-inflammatory therapy. Furthermore, researchers found that Bai could promote the transition of macrophage phenotype from M1 to M2, thereby regulating the progression of inflammation and improving inflammatory symptoms [17–20]. It is expected that Bai may be promising for inducing M2 macrophage polarization in the treatment of OA. However, it has not yet been applied clinically for OA treatment, possibly limited by drug delivery, low solubility, instability and susceptibility to rapid clearance by the body [21]. Therefore, it is necessary to construct an effective drug sustained-release delivery system to improve its bioavailability and therapeutic effect.

Metal-organic frameworks (MOFs) [22–24] are a new type of porous nano-platform with abundant metal sites that have been used as carriers in drug delivery systems [25, 26], biological imaging [27, 28] and chemical sensing [29]. Among them, UIO-66 has been widely used as a drug delivery system due to its high drug-loading capacity, easy functionalization, excellent biocompatibility, stability and biodegradability [30–32]. Furthermore, it possesses the unique structural and functional properties of MOF, which may improve the solubility and stability of loaded drugs. However, MOF has poor cartilage permeability, which limits its application in OA therapy. Modifying the surface of UIO-66 to improve the drug delivery efficiency may be an ideal choice. Folic acid (FA), hyaluronic acid (HA), polypeptides and other compounds have been used to functionalize the surface of MOFs [33, 34]. Li et al. improved the bioavailability by developing a pegylated chitosan-decorated UiO-66 drug carrier [35]. Mozhgan Parsaei et al. synthesized a quercetin-loaded drug carrier using MOF-808-based folate-coupled chitosan (CS-FA), which showed high drug loading and efficient targeting as compared with unmodified MOF-808 [36]. These evidences suggested that modified MOFs are promising drug carriers for targeting and sustainable release.

**Fig. 1:**
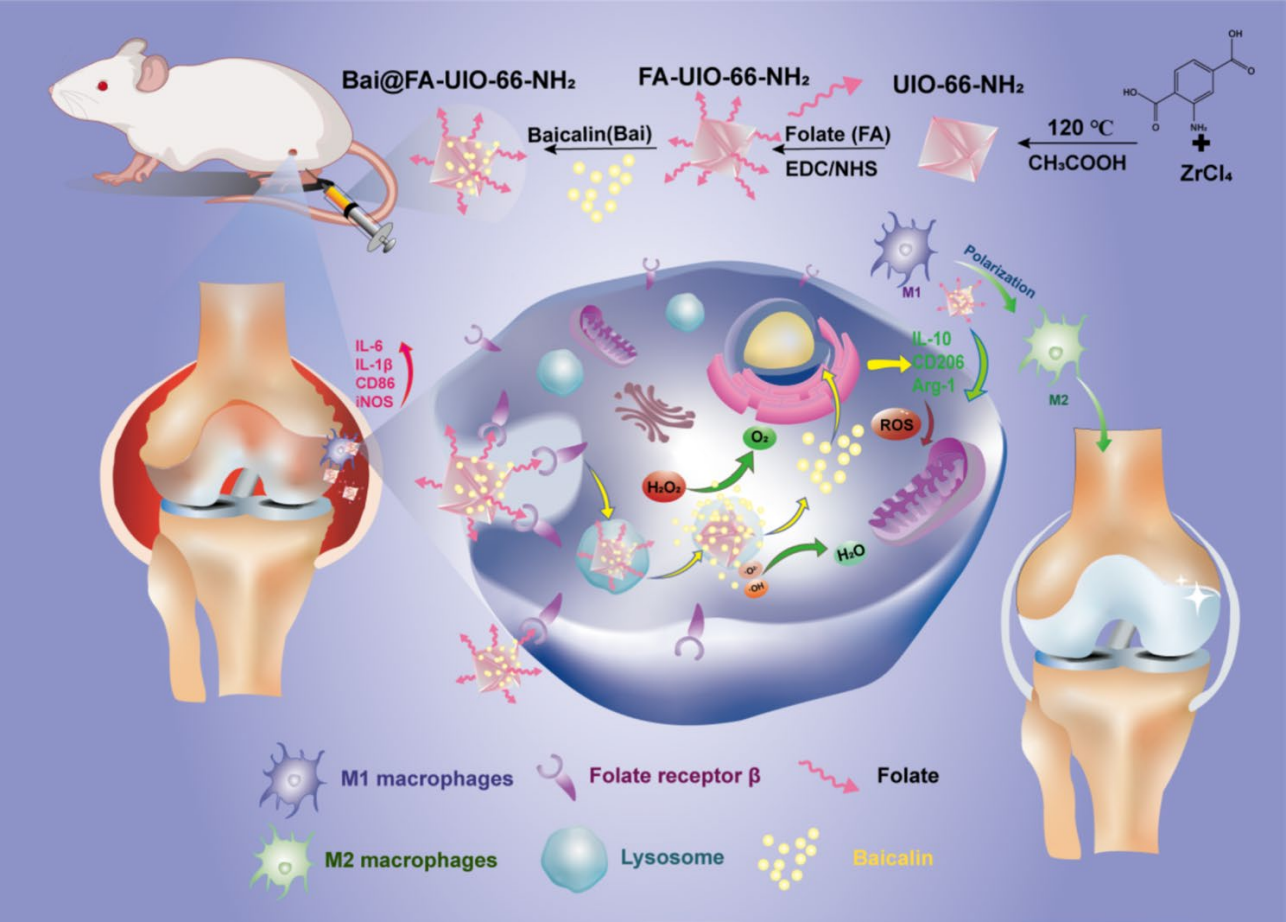
Schematic illustration of Bai@FA-UIO-66-NH_2_ mediated ROS reduction and macrophage polarization regulation for effective treatment of OA. The M1-macrophage targeted drug delivery system Bai@FA-UIO-66-NH_2_ synergistically clears high ROS and promotes the transformation of M1 macrophage to M2 for treating inflammation synovitis

Hence, we developed a novel macrophage-targeting drug delivery system Bai@FA-UIO-66-NH_2_ containing Bai and FA-modified Zr-based organic frameworks to improve drug bioavailability, which acts as a ROS scavenger to regulate macrophage polarization for OA therapy (Fig. 1). In the system, Zr-based MOF exhibited low cytotoxicity and high loading capacity due to its high porosity and Zr^4+^ coordination, and further FA-modification promoted targeted delivery by targeting overexpressed folate receptors of M1 macrophages, leading to Bai accumulation in synovitis joints and enhanced anti-inflammatory activity. In conclusion, this novel drug delivery platform could simultaneously achieve efficient targeted drug delivery and ROS scavenging, providing a therapeutic strategy for OA.

## Materials and methods

### Materials

Baicalin (Bai, 90%), Zirconium (IV) chloride (ZrCl_4_, 94%), 2-aminoterephthalic acid (> 98%), N, N-dimethylformamide (DMF, ACS), CH_3_COOH (ACS), folic acid (FA, ≥ 98%), N-(3-Dimethylaminopropyl)-N′-ethylcarbodiimide hydrochloride (EDC·HCl, 98%), DMSO (≥ 99.9%), PBS buffer (pH = 7.4), HCl (ACS), LPS, N-hydroxysuccinimide (NHS, 98%) and chlorpromazine (CPZ, 95%) were purchased from Aladdin, China. All chemicals were utilized directly without the necessity for additional purification.

### Preparation of UIO-66-NH_2_ NPs

First, add 2.27 mmol ZrCl_4_ and 2.11 mmol 2-amino terephthalic acid to the round bottom flask. N, N-dimethylformamide (DMF) and CH_3_COOH were then added to the above mixture at room temperature in a volume ratio of 100: 3.5, respectively. The above mixture was then put in a high-pressure reaction kettle at 120 °C for continuous reaction. After 24 h, the precipitate obtained by centrifugation (4000 rpm, 10 min) was washed two to three times with methanol before drying overnight at room temperature.

### Synthesis of FA-UIO-66-NH_2_ NPs

FA-UIO-66-NH_2_ was synthesized using an amidation condensation reaction in which the carboxylic acid group of FA reacted with the amino group (-NH_2_) of UIO-66-NH_2_ catalyzed by EDC/NHS. Under light-protected conditions, 10 mg FA, 30 mg EDC·HCl, and 10 mg NHS were activated in DMSO for 2 h. Then, 10 mg UIO-66-NH_2_ nanoparticles were added to the solution mentioned above and allowed to react for 24 h. Finally, the centrifuged precipitates were dialyzed with water to remove any remaining FA, EDC, or NHS. After 72 h of dialysis, the resulting products were collected by centrifugation and named FA-UIO-66-NH_2_.

### Synthesis of Bai@FA-UIO-66-NH_2_ NPs

In a light-protected environment at room temperature, 5 ml of Bai (2 mg/ml) was mixed with 20 ml of FA-UIO-66-NH_2_ (0.1 mg/ml). The mixture was stirred using a magnetic stirrer for 24 h. After the reaction, the mixture was centrifuged at 4000 rpm for 10 min. The isolated lower precipitate was collected and centrifuged three times with deionized water, yielding the ultimate precipitate, Bai@FA-UIO-66-NH_2_.

### Characterization of NPs

The structural characteristics of UIO-66-NH_2_ NPs and FA-UIO-66-NH_2_ NPs were confirmed through a comprehensive analysis using X-ray diffraction (XRD, Rigaku, Japan), Fourier transform infrared spectroscopy (FT-IR, IRAffinity-1 S, Japan) and Ultraviolet and visible spectroscopy (UV-vis, Shimadzu, Japan). Morphological and elemental distribution of the samples were further elucidated using a Scanning electron microscope (SEM, Bruker, Germany), Transmission electron microscope (TEM, Bruker, Germany), X-ray photoelectron spectroscopy (XPS, ESCALAB 250Xi, US) and Energy dispersive X-ray spectrum (EDS). Dynamic light scattering (DLS), Zeta potential and N_2_ isothermal adsorption curve contributed to the characterization of particle size, surface charge and porosity. Additionally, thermal stability was assessed through thermogravimetric analysis (TGA, Star449, Germany).

### Drug loading ratio

The drug-carrying efficacy of FA-UIO-66-NH_2_ NPs for Bai was determined using a centrifugal method. Specifically, Bai and FA-UIO-66-NH_2_, at varying weight ratios, were combined in a certain volume solution and centrifuged. The resulting supernatant was collected, and the unloaded Bai content was measured using a UV-visible spectrophotometer. The drug loading capacity and encapsulation rate of FA-UIO-66-NH_2_ NPs were then calculated. The optimal drug-to-drug ratio was determined to investigate the drug loading performance of FA-UIO-66-NH_2_ nanocarriers for Bai. The applicable calculation formula is as follows:

$$Loading\,efficiency(\% )\, = \,{{{m_{Bai}} - {c_1}{v_1} - {c_2}{v_2}} \over {{m_{FU}}}}\, \times 100$$

$$Encapsulation\,efficiency(\% )\, = \,{{{m_{Bai}} - {c_1}{v_1} - {c_2}{v_2}} \over {{m_{Bai}}}}\, \times 100$$

Among them, *m*_*Bai*_ is the initial input of Bai, *m*_*FU*_ is the initial input of FA-UIO-66-NH_2_, *c*_*1*_ and *c*_*2*_ are the concentrations of Bai in supernatant 1 obtained by the first centrifugation after the reaction and supernatant 2 collected after washing with 5 ml deionized water, *V*_*1*_ and *V2* are the volumes of supernatant 1 and supernatant 2, respectively.

### Drug release rate

To investigate the release kinetics of Bai from the Bai@FA-UIO-66-NH_2_ nanocarriers under various conditions, 5 ml Bai@FA-UIO-66-NH_2_ PBS suspension of different pH was introduced into a dialysis bag. Subsequently, 95 ml of the corresponding PBS buffer solution served as the external dialysis solution. The entire dialysis lasted 48 h and was carried out at room temperature and in the dark. Samples were collected at predetermined intervals, including 0.25 h, 0.5 h, 1.0 h, 2.0 h, 6 h, 8 h, 12 h, 24 h and 48 h. Each sample consisted of 5 mL of dialysis fluid extracted and stored in a 10 mL EP tube, that was kept away from light. Simultaneously, an equivalent volume of the corresponding PBS buffer was added to the dialysis fluid, keeping the system’s total volume constant at 100 mL. A UV-visible spectrophotometer with a wavelength of 276 nm was used to measure the dialysis fluid’s absorbance at each time point. Finally, analysis was carried out using the Bai standard regression equation and the formula for calculating the cumulative drug release rate. The formula to calculate the cumulative drug release rate is as follows:

$$\eqalign{ the\,cumulative{\kern 1pt} \,drug\,release\,rate(\% )\, = & \cr & {{{v_e}\sum\nolimits_1^{n - 1} {{c_i} + {c_n}{v_0}} } \over m}\, \times 100 \cr}$$

Where V_e_ is the volume of each sample, V_0_ is the total volume of dialysate, *c*_*i*_ is the concentration of Bai in the samples sampled for the *i* time, *m* is the dosage of Bai, and *n* is the number of sampling times.

### The stability study of the NPs

To assess the stability of the NPs, Bai and Bai@FA-UIO-66-NH_2_ NPs were dispersed at a concentration of 2 mg/mL in various solutions (PBS = 7.2, PBS = 5.4 and DMEM medium). The images of the NPs were photographed and the diameters of Bai@FA-UIO-66-NH_2_ were measured by DLS at 0, 1,3,5 and 7 d.

### Hemolysis test

To analyze the hemocompatibility of the NPs, the hemolysis test was performed with fresh whole blood of SD rats. First, the whole blood of the rats was collected through the abdominal aorta with an anticoagulant tube. After five minutes of centrifuging the collected whole blood at 1000 rpm, the top layer was discarded. The underlying red blood cells were repeatedly washed with normal saline (NS) until the supernatant was colorless, and then prepared into a 2% w/v red blood cell suspension with normal saline (NS). 2 mL of NPs (FA-UIO-66-NH_2_, Bai and Bai@FA-UIO-66-NH_2_) with the same concentration (0.1 mg/ml) were incubated with an equal volume of the diluted red blood cell suspension at 37℃ for 2 h. Furthermore, a diluted red blood cell suspension was combined with an equal volume of distilled water and normal saline, which were designated as the positive and negative controls, respectively. Lastly, a UV-vis spectrophotometer was used to measure each sample’s hemoglobin release at 540 nm. The hemolysis percentage was calculated as follows:

4 $$\eqalign{ Hemolysis(\% )\, = & \cr & {{(A{b_{sample}} - A{b_{negative\,control}})} \over {(A{b_{positive\,control}} - A{b_{negative\,control}}}} \times 100 \cr}$$

### ROS scavenging ability of NPs

To assess the ROS-scavenging abilities of the NPs, the SOD activity assay kit (Sigma-Aldrich, USA), CAT activity assay kit (Beyotime, China),  ABTS detection kit (Solarbio, China) and ·OH detection kit (Solarbio, China) were used following the kit instructions. The absorbance was measured at 450 nm using a microplate reader (Thermofisher, USA) and calculated the SOD enzyme activity of the various samples. Furthermore, the absorbance was respectively measured at 550 and 520 nm and 405 nm for evaluating CAT-like activity, ·OH detection ability and total antioxidant capacity.

### Cell culture

The most popular in vitro model for studying inflammation and identifying anti-inflammatory active agents is the RAW264.7 cell line. The RAW 264.7 cells used in this study were commercially purchased from the American Type Culture Specimen (ATCC, USA). All cells were respectively cultured in Dulbecco-modified Eagle medium. The cultured medium was replaced every 2 days. Cells were passaged when reached 80–90% and collected for additional study.

### Cell cytotoxicity assay

The cytotoxicity of Bai and NPs on RAW264.7 macrophages was assessed using the Cell Counting kit-8 (CCK-8, Biosharp). Briefly, RAW264.7 macrophages were cultured in a 96-well plate for 24 h. The cells were then subjected to varying concentrations of Bai, FA-UIO-66-NH_2_ and Bai@FA-UIO-66-NH_2_ for an additional 24 h. Subsequently, 100 µL DMEM medium solution containing 10% CCK-8 was added, and the samples were co-cultured for 2 h. The resulting absorbance at 450 nm was measured using a full-wavelength microplate reader (Thermo Scientific, USA) and the cell viability was calculated. Furthermore, RAW264.7 cells were pre-induced with LPS (10 ng/mL) to induce the M1 phenotype of macrophages. The subsequent investigation into the in vitro cytotoxicity of Bai@FA-UIO-66-NH_2_ under OA conditions followed the aforementioned procedure.

### Live and dead staining assay

The Calcein-AM/PI cell staining reagents (Beyotime, China) were used to investigate the biocompatibility. First, RAW264.7 cells (2 × 10^5^ cells) were subjected to co-cultured with Bai and different NPs for an additional 24 h following pre-treatment with LPS (10 ng/ml). Each group received an application of Calcein AM/PI dual dye, which was followed by quantitative analysis, observation, and photography under a fluorescent microscope (Olympus, Japan).

### Intracellular ROS scavenging measurement

The antioxidant capacity was assessed using the Reactive Oxygen Species Assay Kit (Beyotime, China). RAW264.7 cells were initially exposed to LPS (10 ng/ml) at 37℃ for 24 h, then treated with Bai and different NPs for another 24 h. Cells were then co-cultured with 10 µM HPF (maokangbio, China) for ·OH level testing, 5 µM DHE (Beyotime Biotechnology, China) for ·O_2_^−^ level testing, and 20 µM DCF (Solarbio, China) for total ROS level testing. They were then cleaned three times using PBS. Ultimately, an Olympus fluorescent microscope (Japan) was used to measure the relative fluorescent intensity, and Image J was used to perform intensity statistics.

To further quantify intracellular ROS levels, macrophages were treated in the same manner as previously described. BD Biosciences, USA) flow cytometry was used to measure the fluorescence intensity of the collected cells.

### Lysosomal escape and macrophage-targeting

RAW264.7 cells were incubated with Cy5.5-labelled Bai@FA-UIO-66-NH_2_ NPs for 0 h, 2 h, 4 h and 6 h. Cells were then collected, fixed and incubated with Actin-Tracker Green-488 (Beyotime, China) for 30 min, followed by a 15 min DAPI staining. Finally, the material uptake capacity of normal macrophages was observed using a confocal microscope (Leica, Germany).

RAW264.7 cells were cultured for 12 h under various conditions with or without LPS (10 ng/ml), followed by a 1 h culture under conditions with or without free FA, representing conditions -LPS (FA-), -LPS(FA+), +LPS(FA-) and + LPS(FA+). The four groups of cells were then treated with Cy5.5-labelled Bai@FA-UIO-66-NH_2_ NPs for 2 h, respectively. They were then washed three times with PBS, fixed with 4% paraformaldehyde for 15 min, and incubated for 30 min with Actin-Tracker Green-488 (Beyotime, China), followed by a 15-minute DAPI staining. Finally, the cells’ targeting capacity was examined with a confocal microscope (Leica, Germany).

To further investigate the targeting mechanism, LPS-activated macrophages were only treated with chlorpromazine (CPZ, 10 µg/ml) for 1 h, then co-cultured with Cy5.5-labelled Bai@FA-UIO-66-NH_2_ NPs for 2 h, followed by the previously described procedures.

To examine subcellular localization, LPS-induced RAW264.7 cells were co-incubated with Cy5.5-labeled Bai@FA-UIO-66-NH_2_ NPs at various time points. After washing with PBS, the cells were fixed and stained using pre-warmed Lyso-Tracker Green (Beyotime, China) at 37℃ for 1 h. Finally, observations were conducted with a confocal laser scanning microscope (Leica, Germany).

### Quantitative real-time polymerase chain reaction(qRT-PCR)

RAW264.7 cells at a density of 2 × 10^5^ were first seeded in 6-well plates and divided into different treatment groups. The total RNA of RAW264.7 cells was isolated using a total RNA extraction kit (Magen, China) and then qRT PCR with reverse transfer RNA using a qPCR detection system (Roche, Switzerland). The sequences of the required primers are shown in Table S4.

### Immunofluorescence staining

RAW264.7 cells were collected after different treatments and fixed in 4% paraformaldehyde with 3% H_2_O_2_ (Aladdin, China). These cells were sealed with goat serum at room temperature for 30 min. CD206 and iNOS antibodies (Boster Biological, China, 1: 200) were used to incubate cells for 8 h. Staining images were observed using a fluorescence microscope.

### Flow cytometry analysis of macrophage polarization

LPS-induced macrophages (2 × 10^5^ cells) were seeded into a 6-well plate overnight. the subsequent steps were consistent with the previously outlined methods. The collected cells were treated with FITC-labelled iNOS and CD206 antibodies for 30 min, respectively. The surface marker for macrophage was measured by flow cytometry.

### In vivo experiments

#### OA model establishment

Sprague Dawley (SD) rats (170 g, 7–8 weeks old, male) were purchased from Guangxi Medical University Animal Center. In this experiment, the OA model was constructed by anterior cruciate ligament transection (ACLT). After surgery, these rats were randomly assigned to 5 groups (*n* = 6): 0.9% saline, Bai (100 µg/mL), FA-UIO-66-NH_2_ (100 µg/mL) and Bai@FA-UIO-66-NH_2_ (100 µg/mL), and received intraarticular injections with the above formulations (100 µL) once every other day for 8 weeks. Simultaneously, simple incisions of the knee skin and joint capsule were performed on the sham operation group, and no additional treatment was administered. Intra-articular injections were administered once a week, and rat samples from each group were collected 4 and 8 weeks after treatment. In addition, three independent observers were invited to evaluate and score these groups in a double-blind manner using Pelletier’s macro score.

#### In vivo fluorescence imaging

Further study on retention time and in vivo distribution of Bai@FA-UIO-66-NH_2_ NPs in the articular cavity of SD rats using in vivo imaging. The Cy5.5-labeled Bai@FA-UIO-66-NH_2_ NPs were first dispersed in a PBS buffer, forming a solution with a concentration of 100 µg/mL. Next, 100 µL of the solutions were injected into the knee joints of rats. Fluorescence signals were measured and intensity quantified using an in vivo imaging system (AniView 100, BLT, China) at different time intervals (0, 2, 6, 12, 24, 48, 72 h). Various ex vivo organs were also collected after 72 h. The fluorescence intensity was then measured and quantified using identical equipment.

#### Histological staining

SD rats’ knee joints from each group were collected for further evaluation, after decalcification, these samples were paraffin-embedded and stained with hematoxylin-eosin (H&E) (Solarbio, China) and Safranin O-fast green (Solarbio, China). Microscopy was used to collect photographs of these stained samples, which were then evaluated histologically.

#### Immunofluorescence staining

After dewaxing and hydrating, the SD rats’ knee joint slices were added with antigen repair solution and microwaved for 5 min. The slices were removed and naturally cooled to room temperature, then dipped in 3% H_2_O_2_ for 10 min and further sealed with goat serum for another 30 min. The knee slices were then incubated at 4℃ overnight with either iNOS (1: 100) or CD206 (1: 100). After removing the primary antibodies the next day, the FITC-labeled (1: 100) and Cy3-labeled secondary antibodies (1: 100) were incubated, stained and observed under the microscope.

#### Detection of ROS in articular cavity of SD rats

The levels of ROS in the articular cartilage were quantified using a tissue ROS detection kit (Bestbio, China), following the manufacturer’s instructions. In summary, 50 mg of cartilage tissue was homogenized with 1 mL of PBS buffer, then centrifuged at 1,000 revolutions per minute at 4 ℃ for 3 min to obtain the sample for subsequent analysis. Subsequently, 1 µL of the ROS probe (BBoxiProbe) was added to 200 µL of the gathered solutions, then incubated at 37℃ for 30 min. The microplate reader (BioTek, USA) was utilized to measure the absorbance of the mixture, with an excitation wavelength of 488 nm and an emission wavelength of 610 nm.

#### Micro-CT

The rat bone tissues were fixed in 4% formaldehyde overnight, washed with PBS, and scanned using micro-CT (voltage 90 kV, current 70 µA, resolution 10 μm). Each set of scanned images was evaluated at the same threshold to render the 3D structure for each sample. The area beneath the growth plate of the proximal tibia was chosen for three-dimensional histomorphometric analysis to determine bone mineral density (BMD).

#### Statistical analysis

SPP Statistics 22.0 was employed for statistical evaluation. All data are presented as mean ± standard deviation (SD). Each independent experiment was replicated at least three times. One-way ANOVA was used to assess group differences, with *P* < 0.05 indicating statistical significance.

## Results and discussion

### Characterization of NPs

**Fig. 2:**
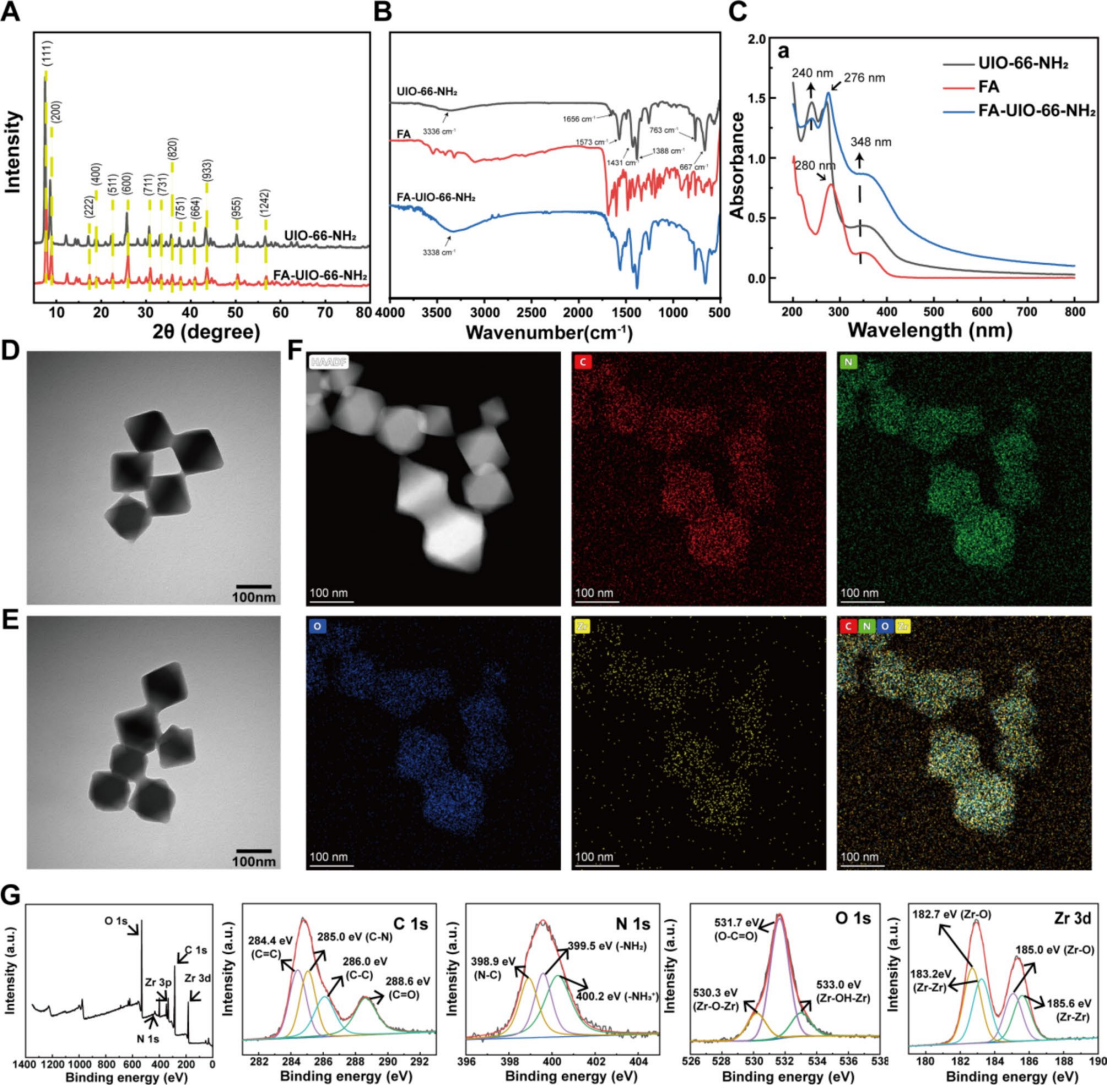
Characterization of NPs. **A**) XRD and **B**) FT-IR and **C**) UV-vis of UIO-66-NH_2_ and FA-UIO-66-NH_2_. **D**) TEM of UIO-66-NH_2_. **E**) TEM of FA-UIO-66-NH_2_. **(F)** Mapping of FA-UIO-66-NH_2_. **G**) XPS of FA-UIO-66-NH_2_

Firstly, the distinct diffraction peaks of the collected UIO-66-NH_2_ (Fig. 2A) identified by XRD analysis were closely aligned with those reported in the literature [37]. The alignment provided strong evidence of the successful synthesis of UIO-66-NH_2_. The XRD pattern of UIO-66-NH_2_ was notable for having fewer impurity peaks and a significantly higher signal intensity, confirming the superior crystallinity and reproducibility of the synthetic material. Moreover, the XRD pattern of FA-UIO-66-NH_2_ is nearly identical to that of its precursor, UIO-66-NH_2_, with only minor variations in the primary characteristic diffraction peaks and their intensities. This observation highlighted the fact that the crosslink between FA and UIO-66-NH_2_ had little effect on the crystal structure or crystallinity of the latter.

The N-H bond in UIO-66-NH_2_ produced an asymmetric vibration absorption peak around 3336 cm^− 1^, as shown by FT-IR results (Fig. 2B). And two characteristic absorption peaks at 763 cm^− 1^ and 667 cm^− 1^, corresponded to the vibration of the Zr-O bond in the internal structure of UIO-66-NH_2_. All absorption peaks were consistent with the previous report, indicating a favorable preparation of UIO-66-NH_2_. In addition, the strong absorption peak around 3338 cm^− 1^ detected in FA-UIO-66-NH_2_ was most likely caused by the N-H bond expansion vibration in the amide bond, suggesting that FA modification was successful [38].

To further verify the successful preparation of FA-UIO-66-NH_2_, we performed UV-vis analysis. As illustrated in Fig. 2C, UIO-66-NH_2_ had the highest absorption peak at 272 nm, while an acromion with a similar peak strength existed at 240 nm. Furthermore, strong absorption peaks around 300 to 440 nm were caused mainly by the amino group in UIO-66-NH_2_, and peaks at 348 nm were consistent with previous literature reports [37], demonstrating the presence of UIO-66-NH_2_. Correspondingly, the UV-vis diagram of FA-UIO-66-NH_2_ contained both characteristic absorption peaks of FA and UIO-66-NH_2_, and the maximum absorption wavelength was shifted to 276 nm, indicating the favorable conjugation of FA and UIO-66-NH_2_.

To examine morphological changes in UIO-66-NH_2_ and FA-UIO-66-NH_2_, we performed TEM (Fig. 2D and E) and SEM (Fig. S1). The results indicated that UIO-66-NH_2_ displayed a 100 to 150 nm size with a distinct octahedral crystal structure, resulting in a smooth, uniform surface morphology and effective dispersion. Moreover, FA-UIO-66-NH_2_ did not differ significantly from UIO-66-NH_2_ in terms of particle size, surface morphology, or dispersibility. This finding implied that the addition of FA did not impair the structural integrity or dispersibility of UIO-66-NH_2_ crystals. For a more in-depth exploration of the elemental distribution in FA-UIO-66-NH_2_, EDS and TEM-mapping analyses were used. The EDS diagram of FA-UIO-66-NH_2_ (Fig. S2) exhibited the presence of C, N, O and Zr. Additional mapping analysis (Fig. 2F) demonstrated a uniform distribution of these elements on the surface of the FA-UIO-66-NH_2_ octahedron. XPS analysis (Fig. S3 and Fig. 2G) was also applied to determine the elemental composition of UIO-66-NH_2_ and FA-UIO-66-NH_2_, which confirmed the presence of C, N, O, and Zr elements. Using XPS PEAKS software to deconvolute the peaks of their elemental maps [38–41], it was discovered that the surface chemical state of FA-UIO-66-NH_2_ remained unchanged by FA modification. However, upon comparing the percentage composition of each element, the N element in UIO-66-NH_2_, post-FA modification, increased significantly from 4.9 to 9.15%. This observation provides further evidence to support the successful grafting of FA onto UIO-66-NH_2_ (Table S1).

TGA was then used to assess the thermal stability of UIO-66-NH_2_ and FA-UIO-66-NH_2_, which revealed that FA modification did not significantly affect the thermal stability of UIO-66-NH_2_ (Fig. S4) [42]. In addition, BET analysis (Fig. S5) revealed standard type II isotherms of UIO-66-NH_2_ and FA-UIO-66-NH_2_, which were primarily mesoporous, with no significant differences in specific surface area, total pore volume, or pore diameter between these two samples after FA modification.

### Characterization of Bai@FA-UIO-66-NH_2_NPs

**Fig. 3:**
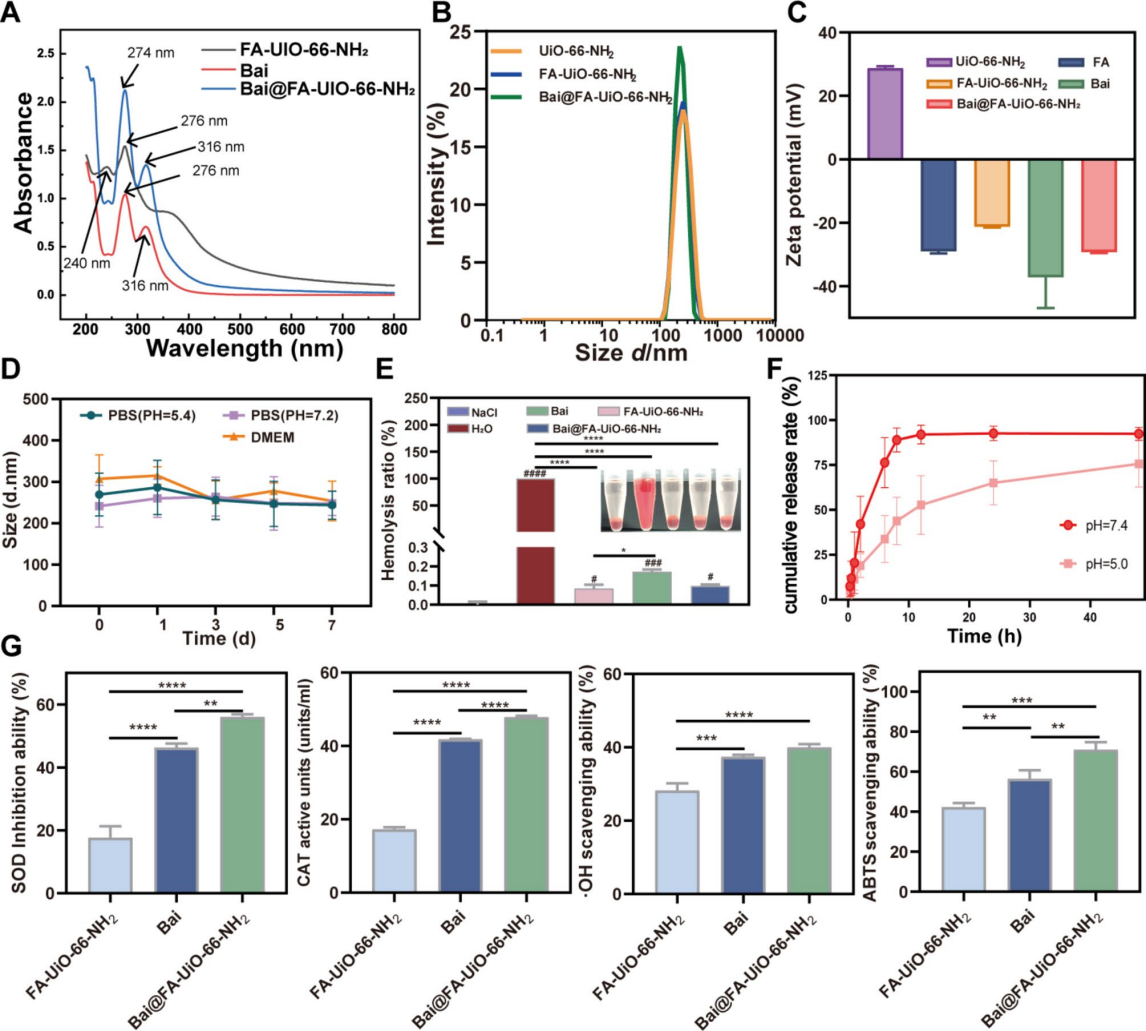
Characterization of Bai@FA-UIO-66-NH_2_ NPs. (**A**) UV-vis of Bai@FA-UIO-66-NH_2_. (**B**) DLS size distribution and (**C**) Zeta potential of FA-UIO-66-NH_2_ and Bai@FA-UIO-66-NH_2_. (**D**) The stability analysis of Bai@FA-UIO-66-NH_2_. (**E**) The hemolysis ratio of Bai, FA-UIO-66-NH_2_ and Bai@FA-UIO-66-NH_2_. (**F**) In vitro cumulative release rate of Bai@FA-UIO-66-NH_2_ with different PBS (pH = 7.4 and pH = 5.0). (**G**) ROS-scavenging effect evaluation. ·OH, ·O_2_^−^ and H_2_O_2_ scavenging ability and the total antioxidant capacity of Bai, FA-UIO-66-NH_2_ and Bai@FA-UIO-66-NH_2_. (*n* = 3, mean ± SD, “#” symbol compared with NaCl group, ^#^*P*＜0.05, ^##^*P*＜0.01, ^*###*^*P*＜0.001, ^####^*P*＜0.0001 and “*” symbol compared between groups, *P＜0.05, **P＜0.01, ***P＜0.001, ****P＜0.0001)

When compared to the UV-vis spectra of free Bai and Bai@FA-UIO-66-NH_2_ NPs, it was found that Bai@FA-UIO-66-NH_2_ had a UV absorption peak similar to that of free Bai around 274 nm and 316 nm, indicating that Bai was successfully loaded on FA-UIO-66-NH_2_ nanocarriers (Fig. 3A). Next, we prepared a series of Bai@FA-UIO-66-NH_2_ NPs with different mass ratios of Bai and FA-UIO-66-NH_2_. when the ratio of Bai and FA-UIO-66-NH_2_ was 7.5:1, the obtained Bai@FA-UIO-66-NH_2_ had the maximum drug encapsulation and loading capacity (Fig. S6) and was chosen for further investigation. Bai@FA-UIO-66-NH_2_ had a slightly smaller average size of 283.4 ± 10.03 nm compared to FA-UIO-66-NH_2_, which had an average diameter of 295 ± 95.18 nm (Fig. 3B), this may be due to hydrophobic interactions between Bai and FA-UIO-66-NH_2_. The changes of the Zeta potential in UIO-66-NH_2_, FA-UIO-66-NH_2_ and Bai@FA-UIO-66-NH_2_ were analyzed in Fig. 3C. In short, the zeta potential of UIO-66-NH_2_ was 28.7 ± 0.6 mV, which increased to -21.2 ± 0.30 mV for FA-UIO-66-NH_2_ after FA modification, and then decreased to -29.3 ± 0.29 mV after Bai encapsulation.

In addition, we evaluated the in vitro stability of Bai and Bai@FA-UIO-66-NH_2_ by monitoring diameter changes in different solutions. The results showed that pure Bai was insoluble, with obvious precipitation at the bottom of the bottle, while Bai@FA-UIO-66-NH_2_ had good dispersion and no obvious aggregation or precipitation of nanoparticles after 7 days of storage in different solutions, demonstrating its stability (Fig. S7). At the same time, the DLS monitoring results showed that within 7 days, the particle diameter of Bai@FA-UIO-66-NH_2_ remained unchanged in PBS = 7.2, PBS = 5.4 or DMEM medium (Fig. 3D). These results verified that Zr-based MOF could enhance the solubility and stability of Bai, this may be related to the fact that encapsulating Bai in MOF could reduce the sensitivity of the drug to the inflammatory environment. In addition, Bai may have ligand bonds or forces with metal ions or ligands in the MOF, thus enhancing drug stability.

showed the hemolysis ratios of FA-UIO-66-NH_2_, Bai and Bai@FA-UIO-66-NH_2_ NPs. After treatment with Bai@FA-UIO-66-NH_2_ NPs, the supernatant of the erythrocyte suspension appeared colorless and transparent, with no signs of erythrocyte rupture observed after centrifugation. The hemolysis rates for FA-UIO-66-NH_2_, Bai and Bai@FA-UIO-66-NH_2_ NPs were all under 0.2%. Bai@FA-UIO-66-NH_2_ NPs exhibited favorable blood compatibility, indicating a promising future application *in vivo*

### In vitro drug loading and releasing properties

The drug release experiment (Fig. 3F) revealed that Bai@FA-UIO-66-NH_2_ exhibited a sudden release effect in a PBS environment with pH = 7.4. In contrast, at pH = 5.0, Bai@FA-UIO-66-NH_2_ demonstrated pH responsiveness, resulting in a sustained-release effect. These findings suggested that Bai@FA-UIO-66-NH_2_ exhibited prolonged slow release in the acidic environment of OA, enabling more efficient and long-lasting OA treatment.

### ROS scavenging ability of Bai@FA-UIO-66-NH_2_

Bai is a flavonoid known for its antioxidant properties and ability to scavenge free radicals [43–45]. In this study, Bai@FA-UIO-66-NH_2_ was tested for its ability to scavenge three major ROS (·OH, ·O_2_^−^ and H_2_O_2_). A preliminary analysis was conducted on the SOD-like activity that scavenges ·O_2_^−^. Figure 3G showed that Bai@FA-UIO-66-NH_2_ eliminated approximately 56% of the ·O_2_^−^, which was significantly higher than FA-UIO-66-NH_2_ and Bai. Following that, the CAT-like activity of Bai@FA-UIO-66-NH_2_ was investigated to monitor H_2_O_2_ decomposition. Similarly, Bai@FA-UIO-66-NH_2_ also exhibited excellent H_2_O_2_ catalytic activity. The order of H_2_O_2_ scavenging ability was Bai@FA-UIO-66-NH_2_> Bai> FA-UIO-66-NH_2_. Compared to Bai and FA-UIO-66-NH_2_, Bai@FA-UIO-66-NH_2_ could effectively scavenge ·OH. Significantly, FA@UIO-66-NH_2_ carrier could remove ·OH due to Zr’s unsaturated coordination bond in UIO-66-NH_2_ [46]. Finally, an ABTS assay kit was applied to detect the total antioxidant capacity of Bai@FA-UIO-66-NH_2_. The results showed that Bai@FA-UIO-66-NH_2_ presented the optimal antioxidant activity, followed by Bai and FA-UIO-66-NH_2_. Thus, Bai@FA-UIO-66-NH_2_ is considered as a ROS scavenger with potential for further research.

### Cell viability assay

Next, LPS-induced RAW264.7 macrophages were used to construct an in vitro OA cell model in this investigation. CCK-8 and Calcein-AM /PI cell staining experiments were performed to evaluate the cytocompatibility of Bai, FA-UIO-66-NH_2_ and Bai@FA-UIO-66-NH_2_. First, the CCK-8 results, shown in Fig. S9, indicated dose-dependent toxic reactions of UIO-66-NH_2_, FA-UIO-66-NH_2_ and Bai towards RAW264.7 cells. Specifically, in Fig. S9a, UIO-66-NH_2_ demonstrated significant proliferation on RAW264.7 macrophages at concentrations ranging from 100 to 200 µg/mL. In comparison to other MOF families [47], the synthesized UIO-66-NH_2_ demonstrated exceptional biocompatibility. This discovery not only improved our understanding of UIO-66-NH_2_ biocompatibility, but also indicated its potential as a promising drug delivery system for OA treatment.

**Fig. 4:**
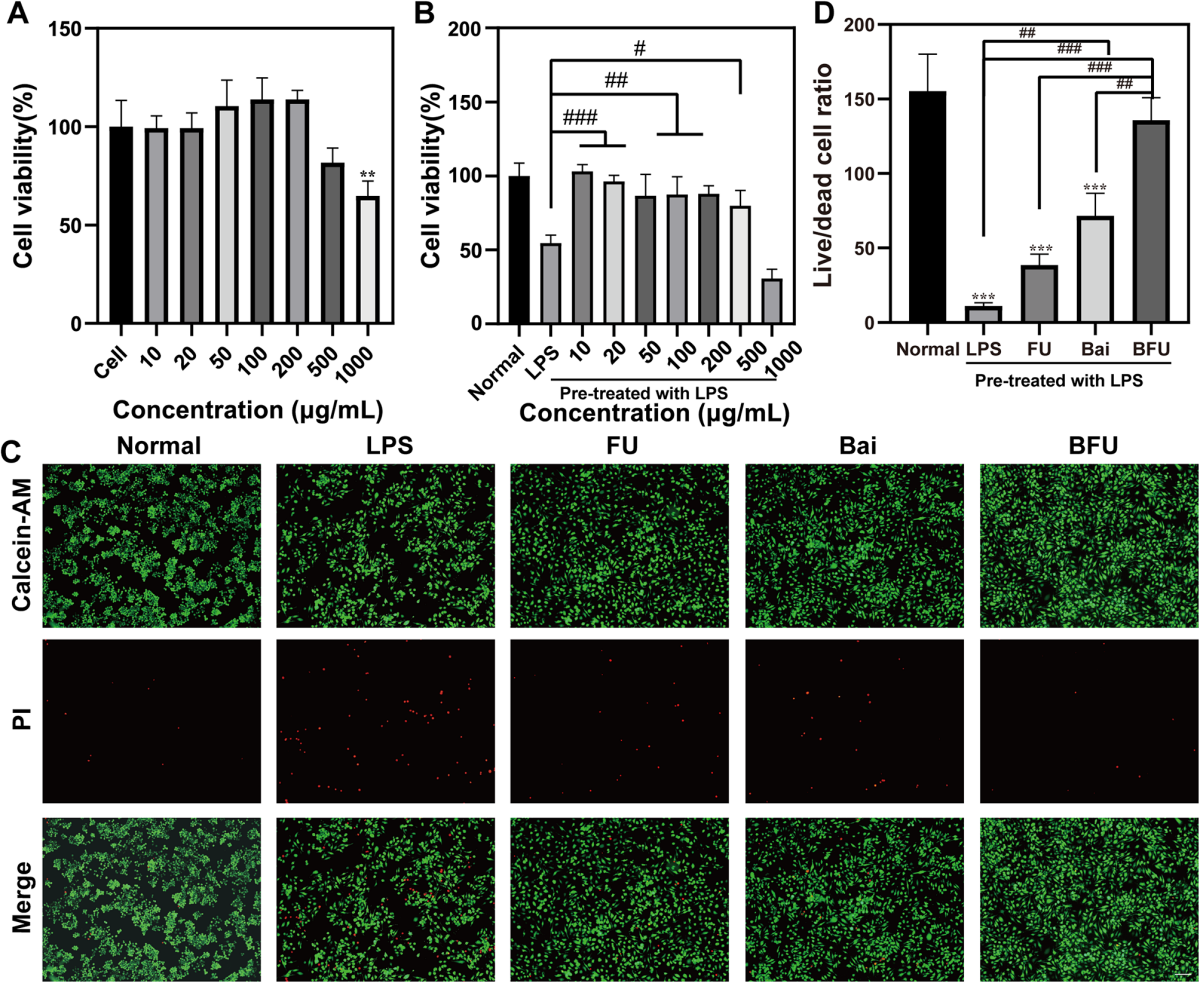
Biocompatibility in vitro. FU (FA-UIO-66-NH_2_), BFU (Bai@FA-UIO-66-NH_2_). CCK-8 of Bai@FA-UIO-66-NH_2_ under without (**A**) and with (**B**) LPS condition. Calcein-AM/PI staining (**C**) and quantification of live/dead cell ratio (**D**). (scale bar = 100 μm, n=3, mean ± SD, “*” symbol compared with normal group, *P＜0.05, **P＜0.01, ***P＜0.001, ****P＜0.0001 and “#” symbol compared between groups, ^#^*P*＜0.05, ^##^*P*＜0.01, ^###^*P*＜0.001, ^####^*P*＜0.0001)

As shown in Fig. S9b, FA-UIO-66-NH_2_ exhibited negligible cytotoxicity to RAW264.7 cells at concentrations below 100 µg/mL. However, in comparison to UIO-66-NH_2_, the cytotoxicity of FA-UIO-66-NH_2_ increased, which was attributed to the introduction of FA. Figure. S9c illustrated that Bai had minimal toxicity to RAW264.7 macrophages at concentrations below 100 µg/mL. At 10 µg/mL, it displayed significant proliferative activity in RAW264.7 macrophages. Additionally, at 200 µg/mL, Bai demonstrated 84.1% activity on RAW264.7 cells. However, above 500 µg/mL, Bai’s cytotoxicity to RAW264.7 macrophages increased significantly. Importantly, after Bai loading, Bai@FA-UIO-66-NH_2_ showed protective effects on RAW264.7 macrophages. No cytotoxic reactions were observed at concentrations between 0 and 200 µg/mL. Even at 50–200 µg/mL, the proliferation-promotion function was found to be unsatisfactory (Fig. 4A). At the same time, after 24 h of LPS intervention, Bai@FA-UIO-66-NH_2_ continued to significantly enhance the vitality of RAW264.7 macrophages (Fig. 4B). When 100 µg/mL Bai@FA-UIO-66-NH_2_ was co-cultured with LPS-induced RAW264.7 for 24 h, the cell viability was increased from 54.5 to 87.4%. Considering all these factors, we have determined that the optimal concentration for Bai@FA-UIO-66-NH_2_ in subsequent experiments will be 100 µg/mL.

Figure 4C illustrated the results of the Calcein-AM /PI cell staining experiment. Compared to the normal group, the LPS group had more dead cells. FA-UIO-66-NH_2_ nanocarriers had a small protective effect on LPS-induced RAW264.7 macrophages, with a live/dead cell ratio of 39.8. Meanwhile, compared to the Bai group alone, the BFU group showed a significant protective effect on LPS-induced RAW264.7 macrophages, with a live/dead cell ratio of 135.7, which was comparable to the normal RAW264.7 cell group (155.2). These findings highlighted the superior protective response of the Bai@FA-UIO-66-NH_2_ treatment against inflammation-induced macrophages.

### In vitro ROS-scavenging measurement

**Fig. 5:**
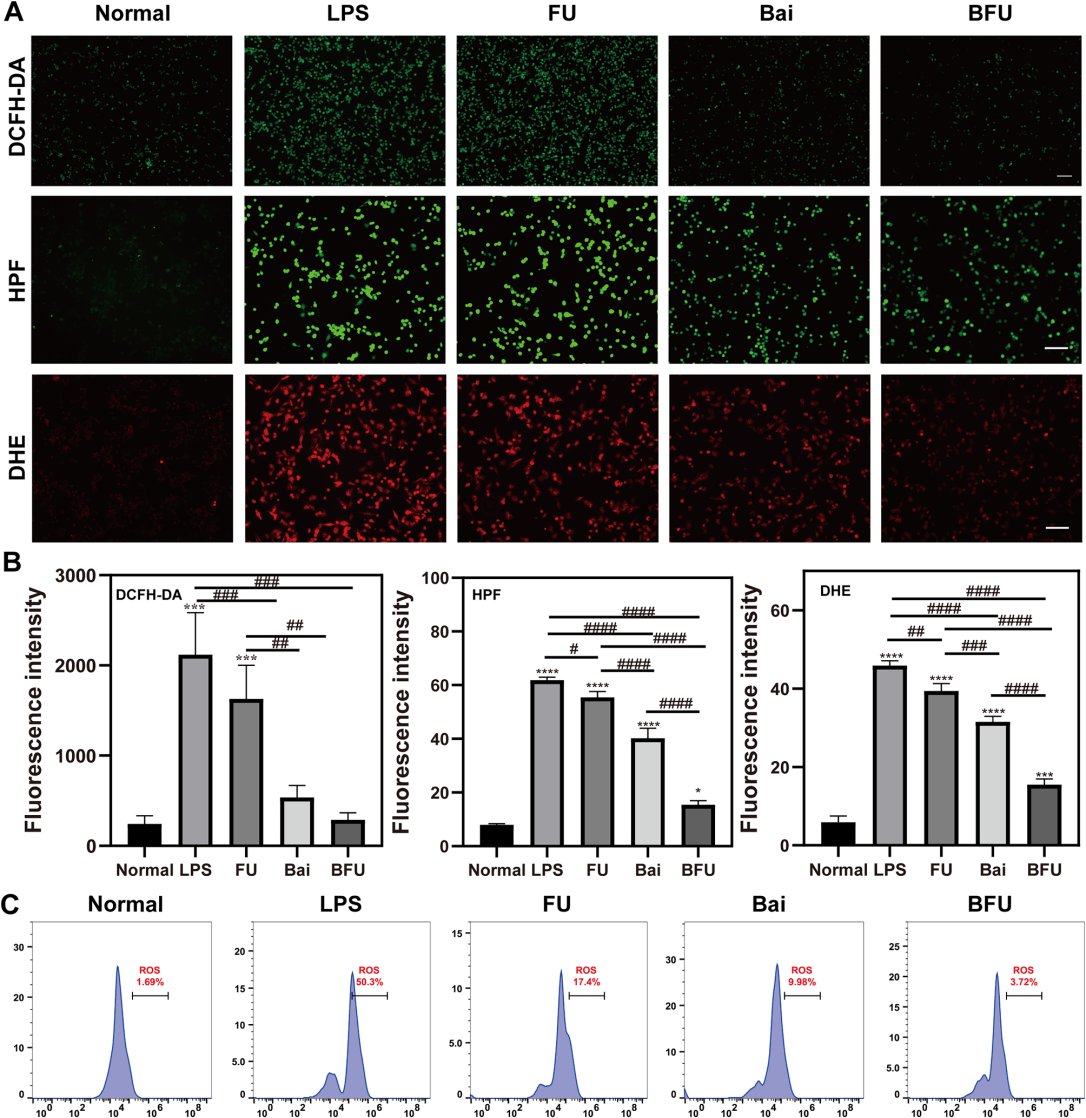
Antioxidant capacity in cellular levels. FU (FA-UIO-66-NH_2_), BFU (Bai@FA-UIO-66-NH_2_). (**A**) Intracellular total ROS (DCF-DA), ·O_2_^−^ (DHE) and ·OH (HPF) levels by fluorescence staining and (**B**) the corresponding quantification of fluorescent intensity. (**C**) Flow cytometry characterization of intracellular total ROS levels. (scale bar = 100 μm, *n* = 3, mean ± SD, “*” symbol compared with normal group, *P < 0.05, **P < 0.01, ***P < 0.001, ****P < 0.0001 and “^#^” symbol compared between groups, ^#^*P *< 0.05, ^##^*P *< 0.01, ^###^*P *< 0.001, ^####^*P *< 0.0001)

Based on previous findings and discussions, Bai@FA-UIO-66-NH_2_ exhibited efficient antioxidant and ROS scavenging ability in vitro. We further assessed the levels of ·O_2_^−^, ·OH and H_2_O_2_ in LPS-induced macrophages treated with Bai, FA-UIO-66-NH_2_ and Bai@FA-UIO-66-NH_2_. DCFH-DA fluorescent probes were first used to investigate the NPs’ ability to decompose H_2_O_2_. As shown in Fig. 5A, compared with the normal group, strong green fluorescence was monitored in RAW264.7 cells stimulated with LPS, confirming ROS overexpression. In contrast, treatment with Bai@FA-UIO-66-NH_2_ significantly reduced intracellular ROS levels. Moreover, FA-UIO-66-NH_2_ stimulus effectively eliminated intracellular LPS-induced ROS overexpression. DHE and HPF fluorescein probes detected intracellular ·O_2_^−^ and ·OH levels, which showed similar trends. The above results were further validated by flow cytometry.

### In vitro cellular uptake and targeting

**Fig. 6:**
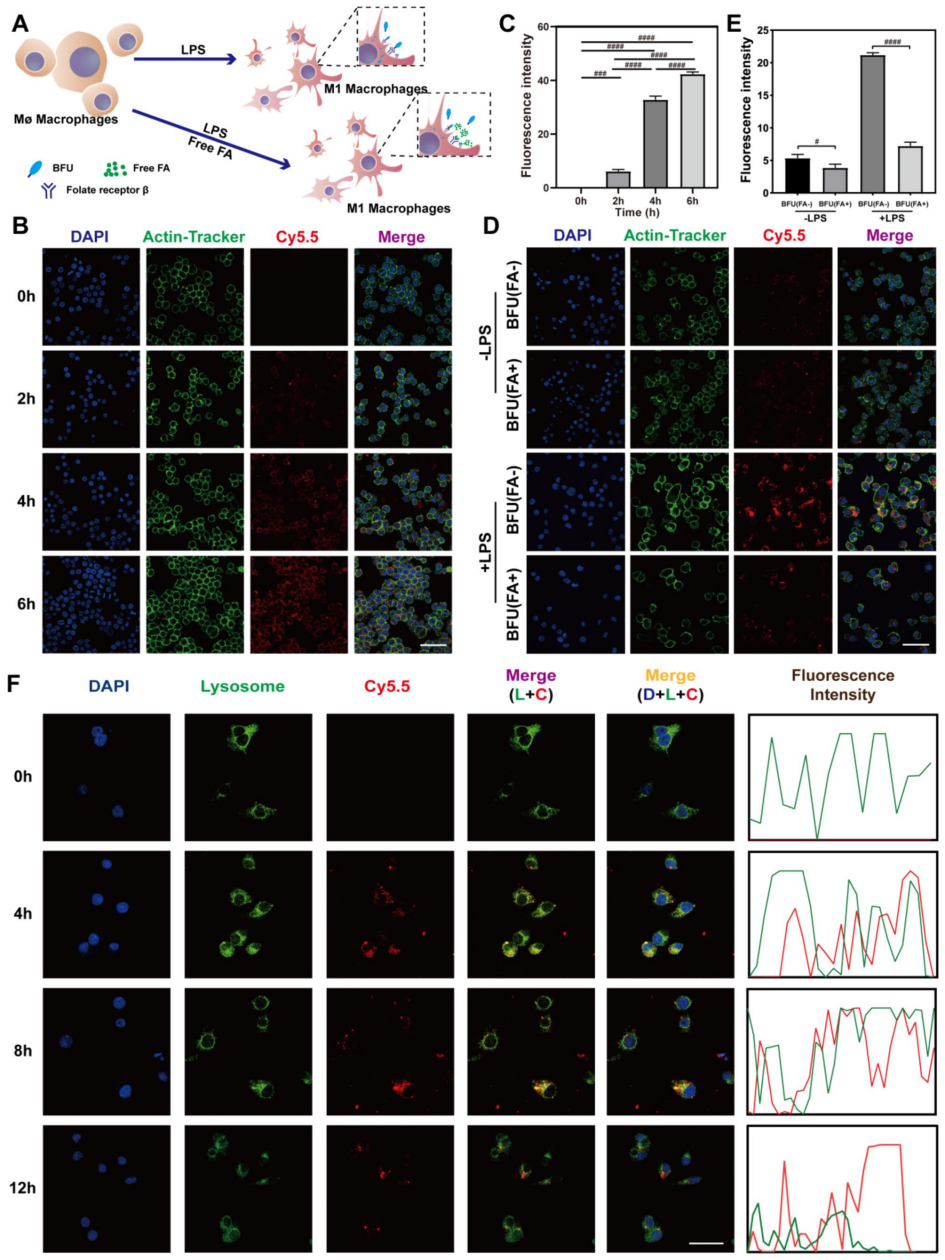
Bai@FA-UIO-66-NH_2_targeting M1 macrophages and lysosomal escape. BFU (Bai@FA-UIO-66-NH_2_). (**A**) Schematic representation of Bai@FA-UIO-66-NH_2_ specificity recognizing LPS-stimulated RAW264.7 cells by binding to highly FR-β on the macrophage. (**B**) Representative pictures of Cy5.5-labeled Bai@FA-UIO-66-NH_2_ uptake by untreated RAW264.7 macrophages and the corresponding fluorescence intensity (**C**) (scale bar = 50 μm). (**D**) Fluorescent pictures of RAW264.7 cells (with or without LPS treatment) treated with Bai@FA-UIO-66-NH_2_ (with or without free FA pre-treatment) and the corresponding fluorescence intensity (**E**) (scale bar = 50 μm). F) Images obtained through CLSM illustrate the co-incubation of LPS-induced inflammatory macrophages with Cy5.5-labelled Bai@FA-UIO-66-NH_2_ over different time intervals. (scale bar = 75 μm*, n* = 3, mean ± SD, “^#^” symbol compared between groups, ^#^*P* < 0.05, ^##^*P *< 0.01, ^###^*P *< 0.001, ^####^*P *< 0.0001)

To assess and confirm Bai@FA-UIO-66-NH_2_’s targeting efficacy on macrophages, we conducted a thorough investigation. Our initial goal was to investigate the effect of untreated RAW264.7 cells on Bai@FA-UIO-66-NH_2_ uptake. Cy5.5-labeled Bai@FA-UIO-66-NH_2_ was administered to untreated RAW264.7 cells for 0, 2, 4 and 6 h. The findings, illustrated in Fig. 6B-C, demonstrated that Cy5.5-labeled Bai@FA-UIO-66-NH_2_ exhibited subtle red fluorescence at 2 h intervals, which gradually increased throughout the incubation period, peaking at 6 h. For a more robust comparative analysis, we choose a 2-hour incubation period in subsequent experiments, distinguishing between conditions with and without LPS induction, with the goal of exploring cellular internalization in greater detail. As shown in Fig. 6D, after 2 h incubation, LPS-stimulated RAW264.7 cells appeared intense red fluorescence throughout the cell, indicating significant cellular uptake. However, the cells untreated with LPS only observed weak red fluorescence in some of the cells. According to a previous study [48], Bai@FA-UIO-66-NH_2_ increased cellular uptake by interacting with the folate receptor beta (FR-β) on LPS-stimulated macrophages, which is overexpressed. Furthermore, pre-treatment with free FA resulted in a significant decrease in the red fluorescence of LPS-stimulated macrophages (Fig. 6E), demonstrating the robust selectivity of Bai@FA-UIO-66-NH_2_ towards these specific macrophages. To further prove that Bai@FA-UIO-66-NH_2_ is internalized through clathrin-mediated endocytosis, chlorpromazine (CPZ) was used to pretreat cells, and the results (Fig. S11) showed that CPZ reduced the cell uptake of Bai@FA-UIO-66-NH_2_, which was also consistent with literature reported previously [48].

In addition, Lyso Tracker staining was performed to further track the co-localization of Bai@FA-UIO-66-NH_2_ NPs with lysosomes in the LPS-induced RAW264.7 cell. The results (Fig. 6F) showed that after 2 h incubation, the red fluorescence of the Cy5.5 label co-localized with the green fluorescence of the Lyso Tracker, demonstrating that Bai@FA-UIO-66-NH_2_ could achieve endocytogenesis in RAW264.7 cells. The red fluorescence increased with incubation time, and lysosomal escape began after 8 h and was complete after 12 h.

### Effects of Bai@FA-UIO-66-NH_2_ on LPS-induced macrophages

**Fig. 7:**
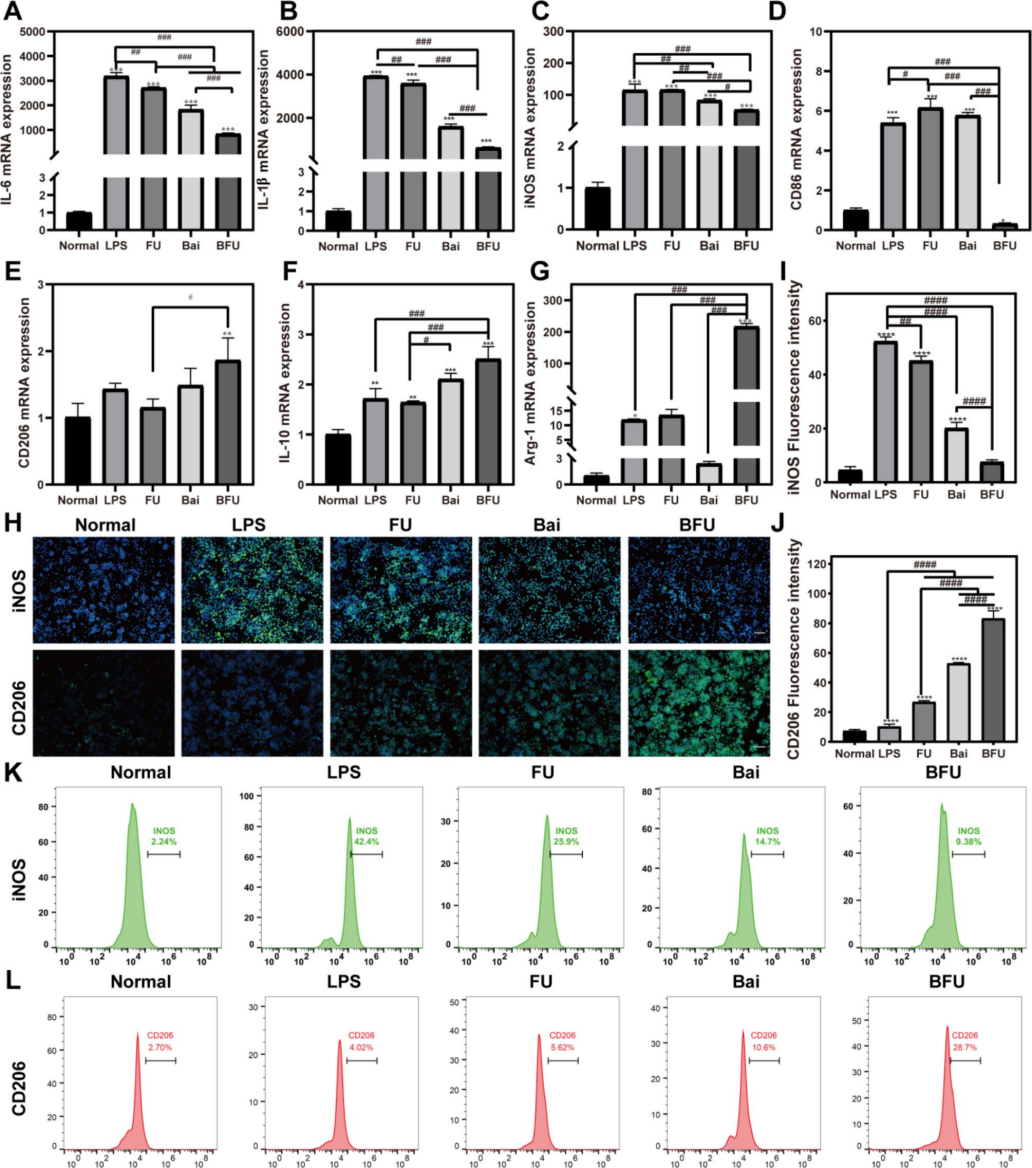
In vitro anti-inflammatory effect produced by driving the polarization of macrophage M1 towards M2. FU (FA-UIO-66-NH_2_), BFU (Bai@FA-UIO-66-NH_2_). (A-G) In vitro mRNA expression of *IL-1β, IL-6, iNOS, Arg-1, CD86, IL-10* and *CD206*. H-J) Immunofluorescence staining of iNOS and CD206 after different treatments. (H) (scale bar = 100 μm) and Corresponding fluorescence intensity quantification (I-J). Flow cytometry analysis of specific marker of M1 macrophage iNOS (K) and M2 macrophage CD206 (L). (*n* = 3, mean ± SD, “*” symbol compared with normal group, *P < 0.05, **P < 0.01, ***P < 0.001, ****P < 0.0001 and “” symbol compared between groups, ^#^*P *< 0.05, ^##^*P *< 0.01, ^###^*P *< 0.001, ^####^*P *< 0.0001)

To assess the anti-inflammatory and the M1-M2 regulatory influence of Bai@FA-UIO-66-NH_2_, we utilized qRT-PCR to measure the expression of inflammation-related (IL-1β and IL-6), M1 polarization-related (CD86 and iNOS), and M2 polarization-related (CD206, IL-10 and Arg-1) genes in RAW264.7 macrophages after treatment. Figure 7A-G showed that, compared to the LPS group, the down-regulation of inflammatory gene expression in the three treatment groups inhibited the inflammatory response of LPS-induced RAW264.7 cells. In addition, in comparison to the LPS and Bai groups, Bai@FA-UIO-66-NH_2_ significantly downregulated M1 polarization genes while upregulating M2-related genes. The order of treatment effectiveness was BFU> Bai> FU.

Next, to further assess the immunomodulatory effect of Bai@FA-UIO-66-NH_2_, we applied immunofluorescence staining to investigate the protein expression of iNOS and CD206 in RAW264.7 macrophages. As shown in Fig. 7H, when compared to other groups, treatment with Bai@FA-UIO-66-NH_2_ on RAW264.7 results in the most evident reduction in the iNOS-positive expression (M1 marker) and elevation in CD206-positive expression (M2 marker). The above results were then verified using flow cytometry. As shown in Fig. 7K and L. Both FU, Bai and BFU groups could inhibit the expression of iNOS on LPS-stimulated macrophages, with iNOS-positive cells in the BFU group being lower 9.38%, indicating that the inhibitory effect on M1-type macrophages was better than that in the Bai group (14.7%). In terms of CD206 expression, the BFU group had a significantly higher percentage of CD206-positive cells, reaching 28.7%, compared to the LPS group’s 4.02%. In summary, Bai@FA-UIO-66-NH_2_ exhibited a remarkable ability to drive macrophage polarization toward the M2 phenotype, surpassing the FU and Bai groups.

In addition, to better reflect the antioxidant capacity of Bai@Fa-UIO-66-NH_2_, we conducted a PCR study using lipoic acid as a control group. Research has shown that lipoic acid is a powerful antioxidant with anti-inflammatory properties derived from its potent free radical scavenging ability, and there have also been recent advances in protecting macrophages [49, 50]. As shown in Fig. S10, Bai@FA-UIO-66-NH_2_ NPs also illustrated excellent antioxidant capacity by up-regulating SOD1 and CAT, when compared to the LPS group. Compared with the lipoic acid group, Bai@FA-UIO-66-NH_2_ NPs were slightly less antioxidant, but their ability to regulate macrophages and reduce inflammation is superior.

### In vivo biodistribution of Bai@FA-UIO-66-NH_2_ NPs

**Fig. 8:**
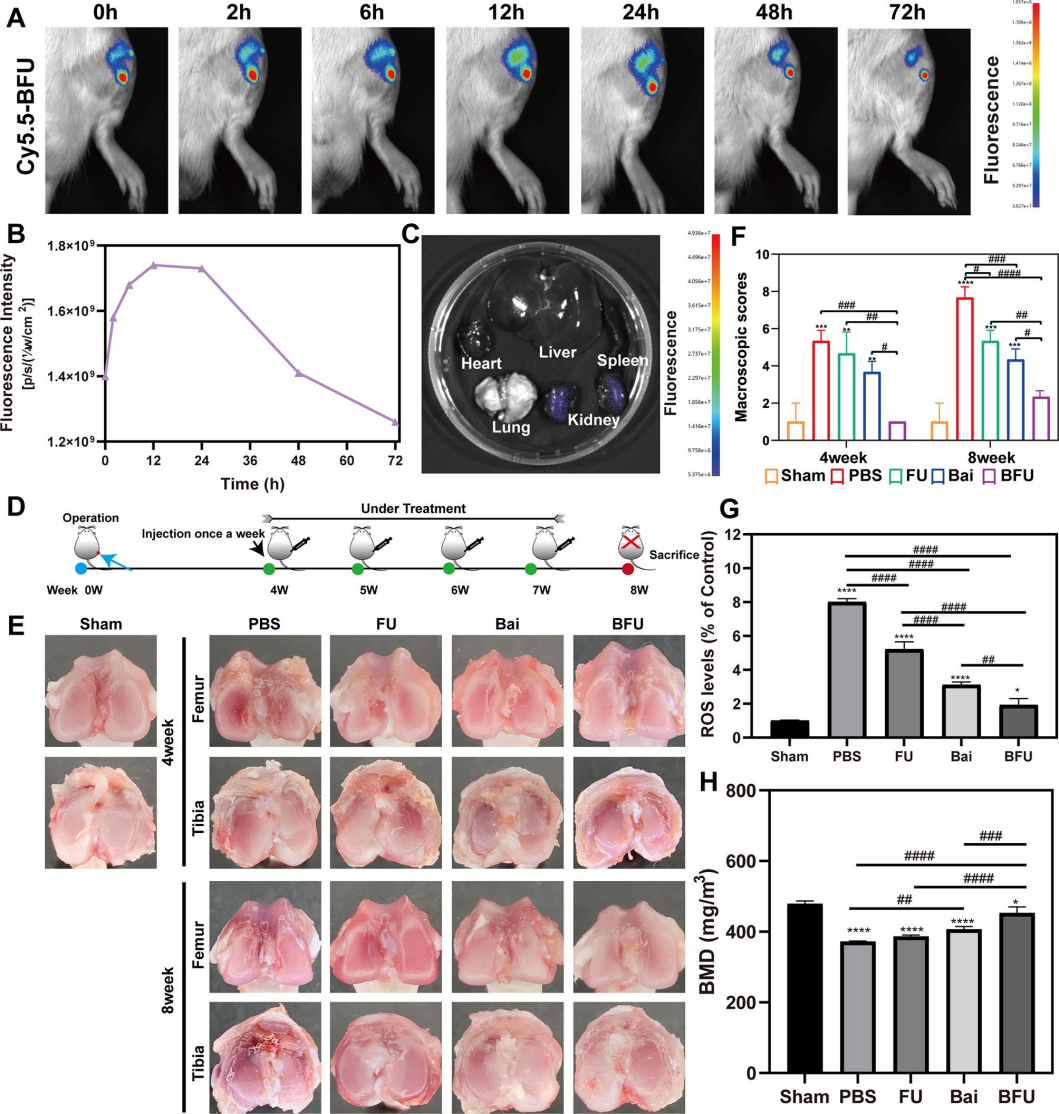
In vivo evaluation of the therapeutic efficacy of Bai@FA-UIO-66-NH _2_NPs in OA SD rats. FU (FA-UIO-66-NH_2_), BFU (Bai@FA-UIO-66-NH_2_). (**A**) Fluorescence images of OA rats following the intra-articular injection of cy5.5-Bai@FA-UIO-66-NH_2_ at different time points and (**B**) corresponding quantitative analysis of fluorescence intensity. (**C**) Fluorescence images of the major organs dissected from OA rats. (**D**) The therapeutic schedule for OA therapy. (**E**) Macroscopic observations and (**F**) the corresponding macroscopic score. (**G**) The levels of ROS in the articular cartilage. (**H**) BMD levels. (*n* = 3, mean ± SD, “*” symbol compared with normal group, *P < 0.05, **P < 0.01, ***P < 0.001, ****P < 0.0001 and “^#^” symbol compared between groups, ^#^*P *< 0.05, ^##^*P *< 0.01, ^###^*P *< 0.001, ^####^*P *< 0.0001)

To further assess the retention time of Bai@FA-UIO-66-NH_2_ in OA rat joints, Cy5.5-labeled Bai@FA-UIO-66-NH_2_ NPs were monitored by an in vivo imaging system. As shown in Fig. 8A, fluorescence intensity gradually increased over the first 12 h and peaked at 24 h. The fluorescence signals then decreased sharply over the next 24 h, with a slight fluorescence remaining at 72 h. Furthermore, fluorescence signals were observed in the rats’ kidneys (Fig. 8C), indicating that Bai@FA-UIO-66-NH_2_ could be eliminated via renal routes.

### Bai@FA-UIO-66-NH_2_ attenuated OA progression in SD rats

As shown in Fig. 8E, the PBS-treated group had fissures, fibrillation, and matrix loss at the junction of the femoral condyle and the tibial plateau. After 4 and 8 weeks, FU, Bai, and BFU groups presented various degrees of improvement, including proteoglycan retention, tidemark integrity promotion, and cartilage erosion reduction. Among all of these groups, the BFU group maintained the smoothest and most integrated cartilage structure, while also increasing tissue cellularity and cell cloning. Furthermore, the Mankin score of the BFU group decreased to 4.33 at 4 weeks and 5.67 at 8 weeks, whereas it was 11.33 at 4 weeks and 13.67 at 8 weeks in the PBS group.

Subsequently, the levels of ROS in the articular cartilage were then assessed using a ROS testing kit. At 8 weeks, there was a significant increase in the OA group compared to the sham group, suggesting elevated levels of ROS in the articular cartilage of OA rats. A notable reduction of 63.5% in ROS levels was observed with Bai@FA-UIO-66-NH_2_ NPs treatment, as shown in Fig. 8G. ROS levels increased steadily over time as OA progressed. Nevertheless, Bai@FA-UIO-66-NH_2_ NPs demonstrated the ability to significantly decrease ROS levels, leading to the optimal therapeutic outcome for OA. In addition, Fig. 8H demonstrated that the analysis results of bone mineral density (BMD). Bai elevated BMD levels compared to the PBS group, while the BFU group had higher BMD levels compared to the Bai group alone. This was consistent with previous literature reports that Bai had some effect on osteoporosis, and our drug delivery system enhanced Bai’s therapeutic effect in vivo.

**Fig. 9:**
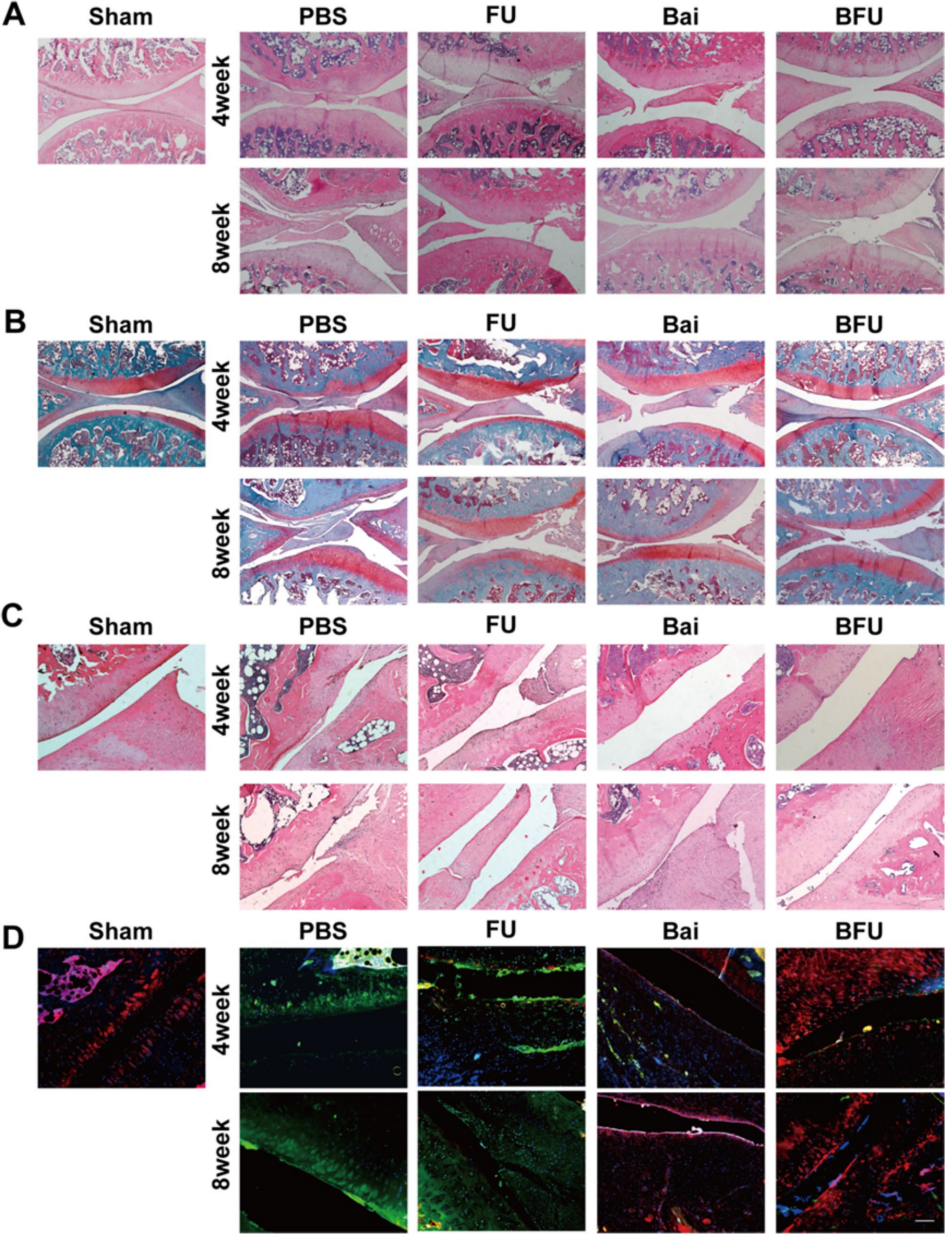
Histological staining evaluation. FU (FA-UIO-66-NH_2_), BFU (Bai@FA-UIO-66-NH_2_). (**A**) H&E staining and (**B**) Safranin O staining of the knee joint of SD rats and (**C**) partial magnified detail (bar = 200 μm). (**D**) Immunofluorescence staining of iNOS/CD206/DAPI of the synovial of SD rats (scale bar = 200 μm)

We further zoomed in the HE results shown in Fig. 9A, demonstrated the presence of cavity inflammation-induced bone erosions in the LPS group, as opposed to the control group. Despite the FU and Bai groups showed promise for cartilage repair, their efficacy was markedly lower than that of the BFU group. Over time, both synovial hyperplasia and cartilage destruction in this group decreased significantly. These above results suggested that Bai@FA-UIO-66-NH_2_ had a favorable therapeutic efficacy against OA.

Moreover, to delve deeper into the potential mechanism underlying the therapeutic impact of Bai@FA-UIO-66-NH_2_ and its potential association with the regulation of macrophage polarization, we conducted histological analysis using immunofluorescence staining for iNOS and CD206 in the synovial of the OA rats joints. Figure 9D showed that the LPS group exhibited more distinct iNOS green fluorescence than the control group, implying a higher proportion of M1 macrophages than M2 macrophages in OA rats’ synovial tissue. This conclusion was also consistent with that reported [10]. Following the treatment with Bai@FA-UIO-66-NH_2_, the green fluorescence area of iNOS decreased while the red fluorescence of CD206 increased. These results showed that M2 macrophage differentiation was enhanced and M1 was inhibited in macrophage polarization after Bai@FA-UIO-66-NH_2_ treatment.

Furthermore, to address biosafety concerns, the in vivo cytotoxicity of Bai@FA-UIO-66-NH_2_ NPs after treatment was investigated via histological analysis. Figure. S13 revealed no discernible tissue damage or lesions in vital organs such as the heart, liver, spleen, lung, and kidney. In conclusion, Bai@FA-UIO-66-NH_2_ has emerged as a promising nano-drug loading platform with numerous applications.

## Conclusion

In summary, we developed an M1 macrophage-targeted Bai@FA-UIO-66-NH_2_ drug release system that could alleviate inflammation by clearing ROS and regulating the polarization of M1 to M2 macrophages for effective OA therapy. Bai@FA-UIO-66-NH_2_ exhibited strong protection against LPS-induced macrophages and had a higher antioxidant capacity than Bai. More importantly, FA actively guided the Bai@FA-UIO-66-NH_2_ nano-system into M1 macrophages, resulting in increased Bai accumulation at the target site, which contributed to a more pronounced alleviation of arthritis inflammation in OA rats. Therefore, we can envisage that Bai@FA-UIO-66-NH_2_ would be a promising therapeutic approach for OA targeting M1 macrophages.

### Electronic supplementary material

Below is the link to the electronic supplementary material.

Supplementary Material 1

## Data Availability

Data is provided within the manuscript or supplementary information files.
