# Supplementary material for: Baicalin nanodelivery system based on functionalized metal-organic framework for targeted therapy of osteoarthritis by modulating macrophage polarization
Source: J Nanobiotechnology. 2024 May 9;22:221. doi: 10.1186/s12951-024-02494-5 (PMC11080297; doi:10.1186/s12951-024-02494-5)
Supplement: Supplementary file 1 — Supplementary Material 1 [file 12951_2024_2494_MOESM1_ESM.docx]

**Supporting Information**

Baicalin nanodelivery system based on functionalized metal-organic framework for targeted therapy of osteoarthritis by modulating macrophage polarization

Lanli Huang ^a, d, 1^, Yi Yao ^a, b, 1^, Zhuren Ruan ^c, 1^, Shengqing Zhang ^a^, Xianjing Feng ^d^, Chun Lu ^e^, Jinmin Zhao ^a, b^, Feiying Yin ^a, b, *^, Cunwei Cao ^c, *^, Li Zheng ^a, b, *^

^a^ Guangxi Engineering Center in Biomedical Material for Tissue and Organ Regeneration, Collaborative Innovation Centre of Regenerative Medicine and Medical BioResource Development and Application Co-constructed By the Province and Ministry, Guangxi Key Laboratory of Regenerative Medicine, The First Affiliated Hospital of Guangxi Medical University, Nanning, 530021, China.

^b^ Life Sciences Institute, Guangxi Medical University, Nanning, 530021, China.

^c^ Department of Dermatology and Venereology, The First Affiliated Hospital of Guangxi Medical University, Nanning, Guangxi, China.

^d^ Pharmaceutical College, Guangxi Medical University, Nanning, 530021, China.

^e^ School of Materials and Environment, Guangxi Minzu University, Nanning, 53000, China.

^1^ The authors contributed equally.

*Corresponding author: [yinfeiying@yeah.net](mailto:yinfeiying@yeah.net), [caocunwei@yeah.net](mailto:caocunwei@yeah.net), [zhengli224@163.com](mailto:zhengli224@163.com).


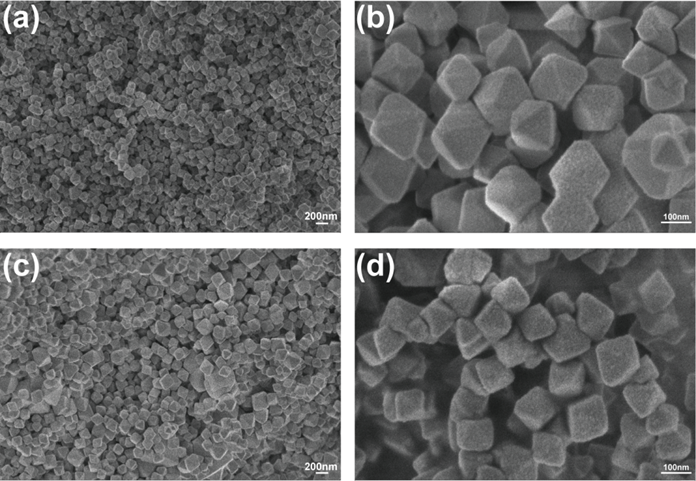


**Figure. S1** SEM of UIO-66-NH_2_ **(a,b)** and FA-UIO-66-NH_2_ **(c,d)** under different multiple.


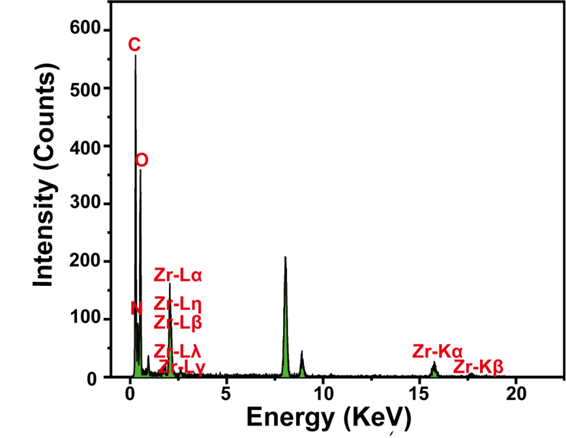


**Figure. S2.** EDS diagram of FA-UIO-66-NH_2_.


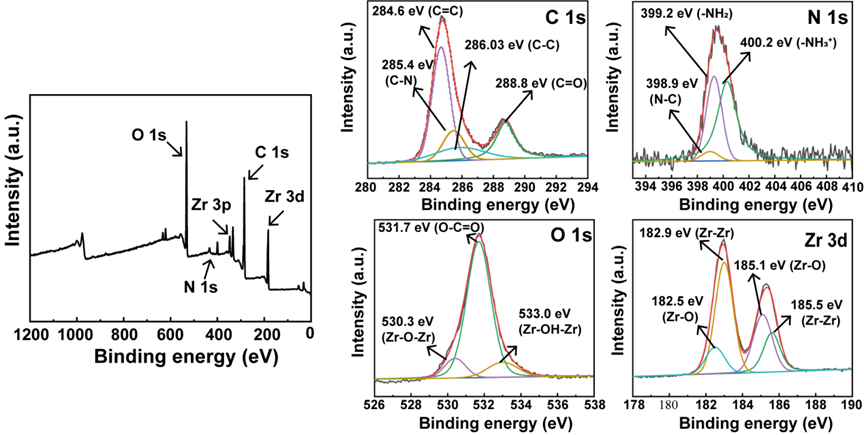


**Figure. S3.** XPS of UIO-66-NH_2_.


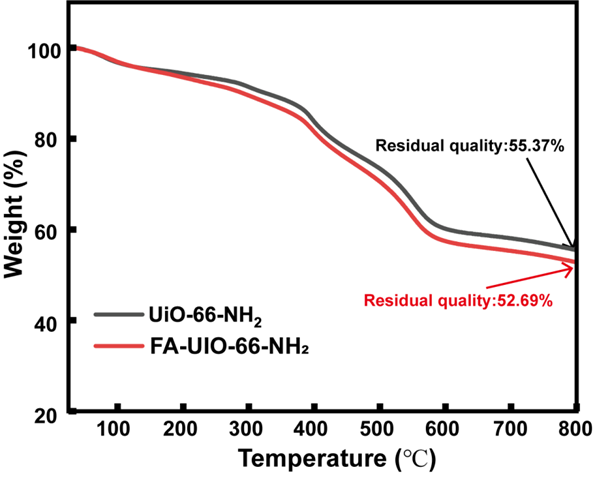


**Figure. S4.** TGA of UIO-66-NH_2_ and FA-UIO-66-NH_2_.


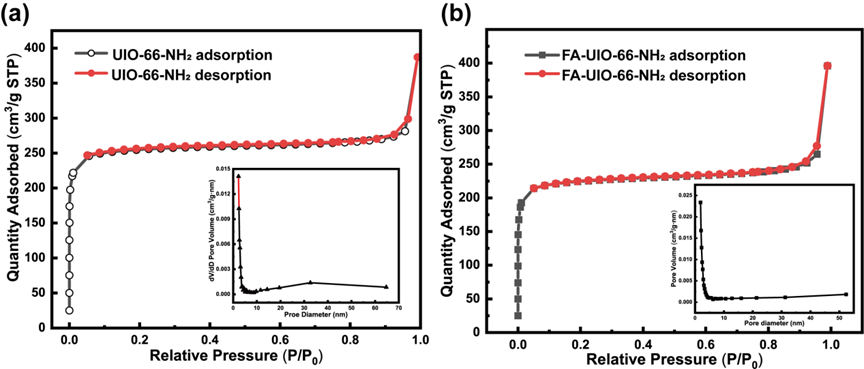


**Figure. S5**. BET of UIO-66-NH_2_ **(a)** and FA-UIO-66-NH_2_ **(b).**


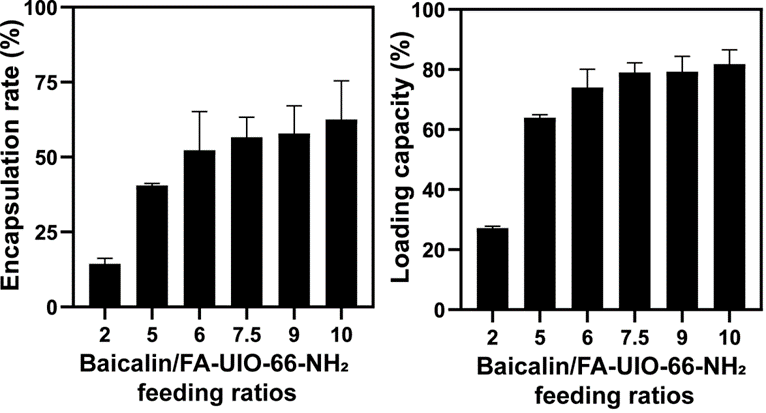


**Figure. S6.** Encapsulation rate **(a)** and loading capacity **(b)** of FA-UIO-66-NH_2_.


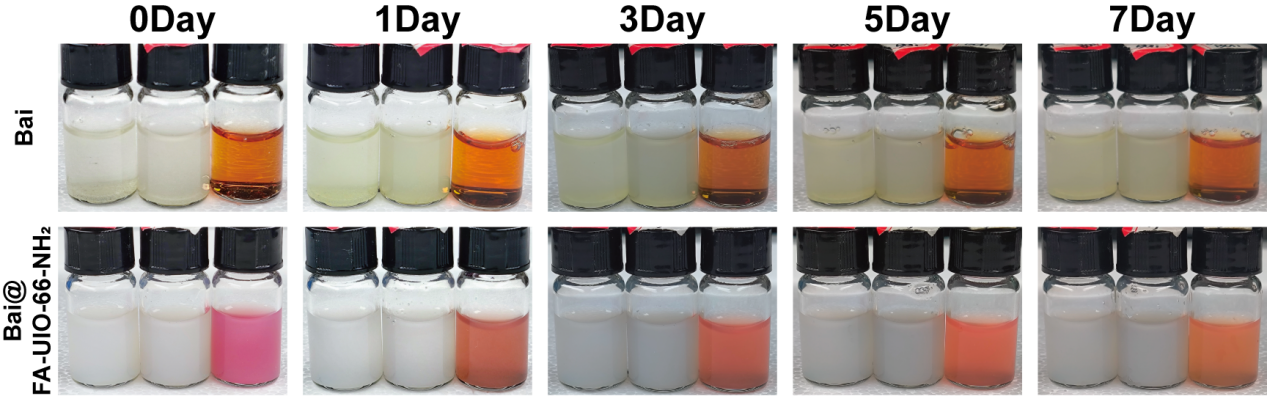


**Figure. S7.** Representative pictures of Bai and Bai@FA-UIO-66-NH_2_ in different solutions at 0, 1, 3, 5 and 7 day.


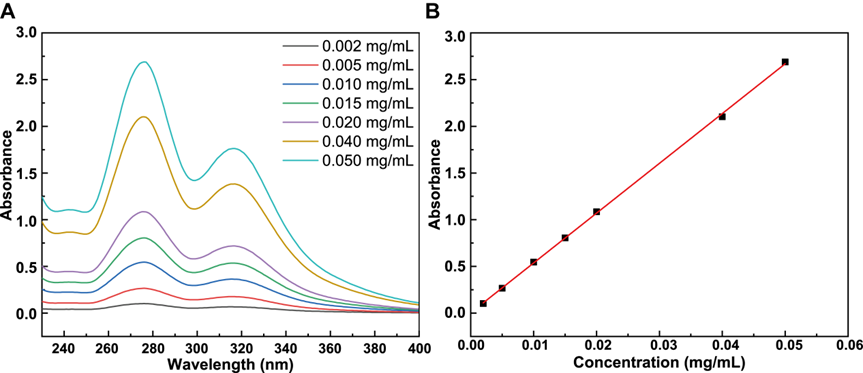


**Figure. S8.** UV-vis spectrums **(A)** and concentration absorbance standard curve of Baiclain **(B)**.


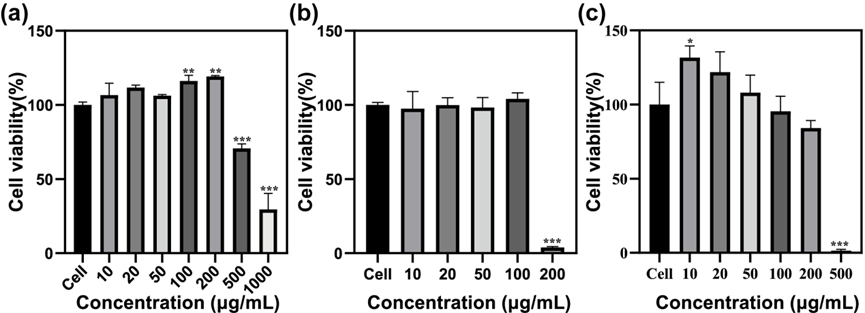


**Figure. S9.** CCK-8 of UIO-66-NH_2_ **(a)**, FA-UIO-66-NH_2_ **(b)** and Bai **(c)**. “^*^” symbol compared between cell and other groups, ^*^*P*＜0.05, ^**^*P*＜0.01, ^***^*P*＜0.001, ^****^*P*＜0.0001).
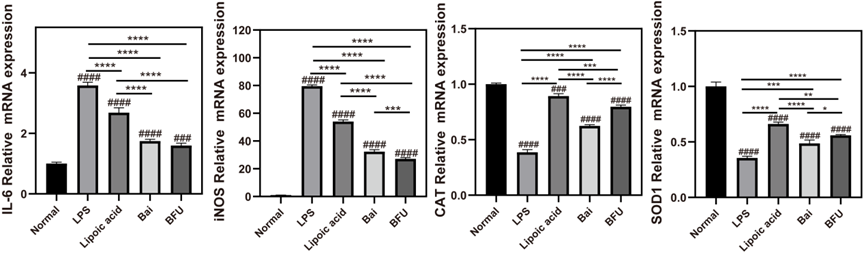


**Figure. S10.** In vitro mRNA expression of IL-6, iNOS, CAT and SOD1. BFU (Bai@FA-UIO-66-NH_2_). (n=3, mean ± SD, “^#^” symbol compared with normal group, ^#^*P*＜0.05, ^##^*P*＜0.01, ^###^*P*＜0.001, ^####^*P*＜0.0001 and “^*^” symbol compared between groups, ^*^*P*＜0.05, ^**^*P*＜0.01, ^***^*P*＜0.001, ^****^*P*＜0.0001).


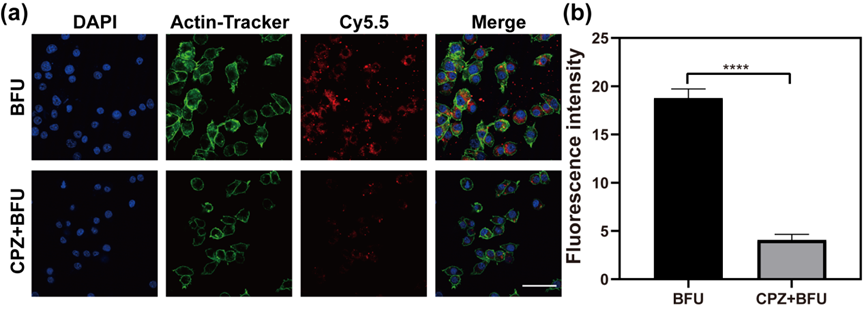


**Figure. S11.** Chlorpromazine (CPZ) interfered the uptake of BFU by RAW264.7 cells. FU (FA-UIO-66-NH_2_), BFU (Bai@FA-UIO-66-NH_2_). **(a)** Fluorescence image and the corresponding fluorescence intensity **(b)**. (scale bar =50 μm). “^*^” symbol compared two groups, ^****^*P*＜0.0001).


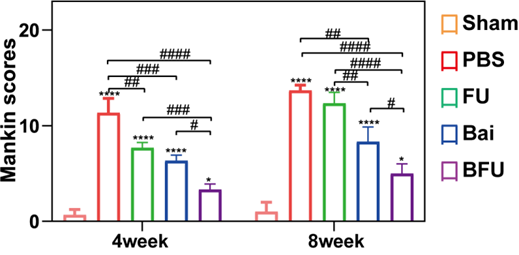


**Figure. S12.** Mankin scores. FU (FA-UIO-66-NH_2_), BFU (Bai@FA-UIO-66-NH_2_). (n=3, mean ± SD, “^*^” symbol compared with sham group, ^*^*P*＜0.05, ^**^*P*＜0.01, ^***^*P*＜0.001, ^****^*P*＜0.0001 and “^#^” symbol compared between groups, ^#^*P*＜0.05, ^##^*P*＜0.01, ^###^*P*＜0.001, ^####^*P*＜0.0001).


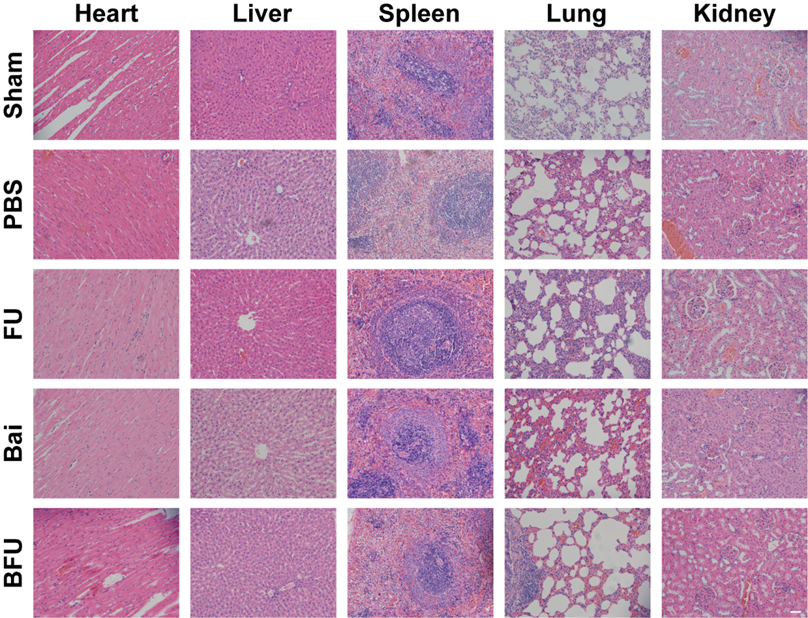


**Figure. S13**. Histological analysis of major organs of SD rats. FU (FA-UIO-66-NH_2_), BFU (Bai@FA-UIO-66-NH_2_). (scale bar =50 μm).

**Table S1.** Percentage change of elements of UIO-66-NH_2_ and FA-UIO-66-NH_2_.

| Sample | C (%) | N (%) | O (%) | Zr (%) |
| --- | --- | --- | --- | --- |
| UIO-66-NH_2_ | 60.15 | 4.9 | 30.79 | 4.16 |
| FA-UIO-66-NH_2_ | 57.69 | 9.15 | 29.27 | 3.89 |

**Table S2.** Surface area, total pore volume and pore size of UIO-66-NH_2_ and FA-UIO-66-NH_2_.

| Sample | S_BET_ (m^2^/g) | Total pore volume [p/p_0_=0.991] | Pore size (nm) |
| --- | --- | --- | --- |
| UIO-66-NH_2_ | 708.15 | 0.5986 | 26.79 |
| FA-UIO-66-NH_2_ | 626.08 | 0.6127 | 25.48 |

**Table S3.** Particle diameter distribution of UIO-66-NH_2_, FA-UIO-66-NH_2_ and Bai@FA-UIO-66-NH_2_ (n=3).

| Sample | Mean particle size ± SD nm | PDI |
| --- | --- | --- |
| UIO-66-NH_2_ | 274.5±17.69 | 0.263±0.030 |
| FA-UIO-66-NH_2_ | 295.4±95.18 | 0.255±0.11 |
| Bai@FA-UIO-66-NH_2_ | 283.4±10.03 | 0.326±0.01 |

The sequence of primers required in the experiment is shown in the following table:

**Table S4** The sequence of primers of RT-PCR.

| Gene | Forward-Primers | Reverse-Primers |
| --- | --- | --- |
| GADPH | ACTTGAAGGGTGGAGCCAAA | GCCCTTCCACAATGCCAAAG |
| IL-1β | TGCCACCTTTTGACAGTGATG | ATGTGCTGCTGCGAGATTTG |
| IL-6 | GGAGCCCACCAAGAACGATAG | GTGAAGTAGGGAAGGCCGTG |
| iNOS | GTTCTCAGCCCAACAATACAAGA | GATGAATTGGATGGTCTTGGTCC |
| CD86 | TGTTTCCGTGGAGACGCAAG | TTGAGCCTTTGTAAATGGGCA |
| IL-10 | TTCAAACAAAGGACCAGC | GGATCATTTCCGATAAGG |
| CD206 | AGGGTGCGGTACACTAACTG | TCTGACTCTGGACACTTGCC |
| Arg-1 | CATATCTGCCAAGGACATCG | GGTCTCTTCCATCACTTTGC |
